# Unconventional secretion of α-Crystallin B requires the Autophagic pathway and is controlled by phosphorylation of its serine 59 residue

**DOI:** 10.1038/s41598-019-53226-x

**Published:** 2019-11-15

**Authors:** M. D’Agostino, G. Scerra, M. Cannata Serio, M. G. Caporaso, S. Bonatti, M. Renna

**Affiliations:** 1 Department of Molecular Medicine and Medical Biotechnologies, University of Naples Federico II, Naples, Italy; 2 Laboratory of Epithelial Biology and Disease, Imagine Institute, Paris, France; 3 Université Paris Descartes-Sorbonne Paris Cité, Imagine Institute, Paris, France

**Keywords:** Macroautophagy, Exocytosis, Secretion, Phosphorylation

## Abstract

α-Crystallin B (CRYAB or HspB5) is a chaperone member of the small heat-shock protein family that prevents aggregation of many cytosolic client proteins by means of its ATP-independent holdase activity. Surprisingly, several reports show that CRYAB exerts a protective role also extracellularly, and it has been recently demonstrated that CRYAB is secreted from human retinal pigment epithelial cells by an unconventional secretion pathway that involves multi-vesicular bodies. Here we show that autophagy is crucial for this unconventional secretion pathway and that phosphorylation at serine 59 residue regulates CRYAB secretion by inhibiting its recruitment to the autophagosomes. In addition, we found that autophagosomes containing CRYAB are not able to fuse with lysosomes. Therefore, CRYAB is capable to highjack and divert autophagosomes toward the exocytic pathway, inhibiting their canonical route leading to the lysosomal compartment. Potential implications of these findings in the context of disease-associated mutant proteins turn-over are discussed.

## Introduction

Proteins that are secreted in an unconventional manner typically lack an N-terminal secretion signal, fail to traffic through the ER and Golgi and do not possess protein modifications indicative of their transit through the secretory pathway^1^. Unconventional protein secretion (UPS) was initially described for interleukin-1β and thioredoxin^2^ and so far it has been proposed for several different proteins^1^. These include insulin-degrading enzymes^3^, FGF-2 and galectin-1^4^, several cytokines such as IL-1, IL-18, IL-33 and IL-1β^5^, nuclear proteins such as high mobility group protein B1 (HMGB1)^6^, the homeoprotein engrailed^7^ and *Dictyostelium discoideum* AcbA^8^ and molecular chaperones such as BAG3^9^. A number of diverse mechanisms for unconventional secretion, including both vesicular and non-vesicular modalities, have been proposed so far, such as: i. direct translocation from the cytoplasm across the plasma membrane by transporters; ii. uptake of proteins into endosomes or lysosomes followed by their fusion with the plasma membrane; iii. plasma membrane blebbing followed by the shedding of extracellular vesicles^10–12^. More recently, it has been shown that also autophagy might be involved and contribute to UPS: indeed, the exosomes-mediated secretion requires first the fusion of autophagosomes with multi-vesicular bodies (MVBs) and then the fusion with the plasma membrane^13,14^. In particular, acyl coenzyme A-binding protein 1 (Acb1) requires autophagy genes as well as the plasma membrane t-SNARE Sso1 for the fusion and release of the Acb1-containing vesicles into the extracellular space^15^.

α-Crystallin B (CRYAB or HspB5) belongs to the group of small heat shock proteins (sHSPs, molecular mass 15–30 kDa). It forms functional oligomers (both homo- and hetero-oligomers), comprising up to 50 subunits and its chaperone activity consists in binding to either cytosolic or transmembrane proteins and preventing their aggregation through an ATP-independent holdase activity^16–19^. Besides the crucial role for vision in retinal cells, as a chaperone protein CRYAB exerts many other important protective functions in other tissues by interacting with the proteasome and the cytoskeleton and also by preventing apoptosis^20,21^. Indeed, malfunctions of CRYAB have been associated to myopathy, neuropathy, ischemia, cataract and cancer^22–25^. In addition, a neuroprotective role has been demonstrated for α-Crystallin B (CRYAB) in the context of Parkinson disease, where it is found as major component of the intracellular Lewy bodies^26^. Intriguingly, a recent report has shown that CRYAB can exert a protective function also in the extracellular compartment, following to its exosome-dependent secretion from polarized human RPE cells, which is mediated by an UPS pathway that involves multi-vesicular-bodies (MVB)^27^. As such, secreted CRYAB has been shown to have a direct role for multiple sclerosis by exerting immuno-modulatory and pro-inflammatory effects^26^.

The required molecular mechanisms and the regulatory steps underlying the secretion pathway of CRYAB are still unknown. In this work, we present evidences that the autophagic pathway is a necessary route to guarantee the unconventional secretion of CRYAB. In addition, we highlight the phosphorylation on a key serine residue of the protein as a crucial negative regulator for its recruitment into autophagosome and consequent secretion.

## Results

### CRYAB is secreted by unconventional pathway from COS-7 cells

In order to study the molecular mechanisms involved in CRYAB secretion, we used the monkey kidney fibroblast COS-7 cell line that endogenously express CRYAB (Fig. S1). To quantify and verify the secretion efficiency of both endogenous and transfected forms of CRYAB, COS-7 cells were transiently transfected with 3xFlag-CRYAB and after an over-night incubation at 37 °C the medium was replaced with DMEM supplemented with 1% FBS and 1% l-Glutamine (Gln). After 6 hours, equal volumes of each medium and lysate were separated by SDS-PAGE and endogenous and over-expressed CRYAB were detected by using a mouse monoclonal anti-CRYAB and anti-FLAG antibodies, respectively. As shown in Fig. S2a, both endogenous and transfected form of CRYAB were detected in culture medium and the efficiency of secretion was quantified as a ratio between extracellular (OUT) and intracellular (IN) fractions. The histogram on the right of the upper panel showed a comparable secretion efficiency of both forms. Hence, and in view of its easier detection as opposed to the endogenous protein, we decided to use the N-terminally 3xFlag-tagged form of CRYAB for the next set of experiments.

To verify that CRYAB is secreted by unconventional secretion, COS-7 cells were transiently transfected with 3xFlag-CRYAB. After 42 hours cells were treated with 5 μg/ml Brefeldin A (BFA) for 6 hours and equal volumes of each medium and lysate were separated by SDS-PAGE and the proteins were detected by immunoblotting. Compared to control cells (BFA-), BFA treatment (BFA+) caused the expected and extensive tubulation/fragmentation of the Golgi complex^28^, as evidenced by GM130 staining shown in Fig. S2b (right panel). Nonetheless, in this condition the secretion of CRYAB was not affected (Fig. S2b, left panels), suggesting that its secretion might not rely on the activity of the canonical secretory pathway.

Finally, to assess whether unconventional secretion of CRYAB could be consequent to the release of exosomes from the cells, COS-7 cells were transiently transfected with 3xFlag-CRYAB and after 48 hours cell culture supernatant was subjected to exosomes isolation by differential centrifugation as previously described^29^. Proteins were then resolved by SDS-PAGE and CRYAB ultimately revealed by using an anti-FLAG antibody. As shown in Fig. S2c, CRYAB was mainly detected in P100 fraction (exosomes), while pre-incubation with the Triton X-100 detergent resulted in a shift of CRYAB in the S100 fraction (i.e., solubilized exosomes), therefore indicating that CRYAB was present in membrane vesicles.

### The autophagic pathway is required for CRYAB unconventional secretion

**Figure 1:**
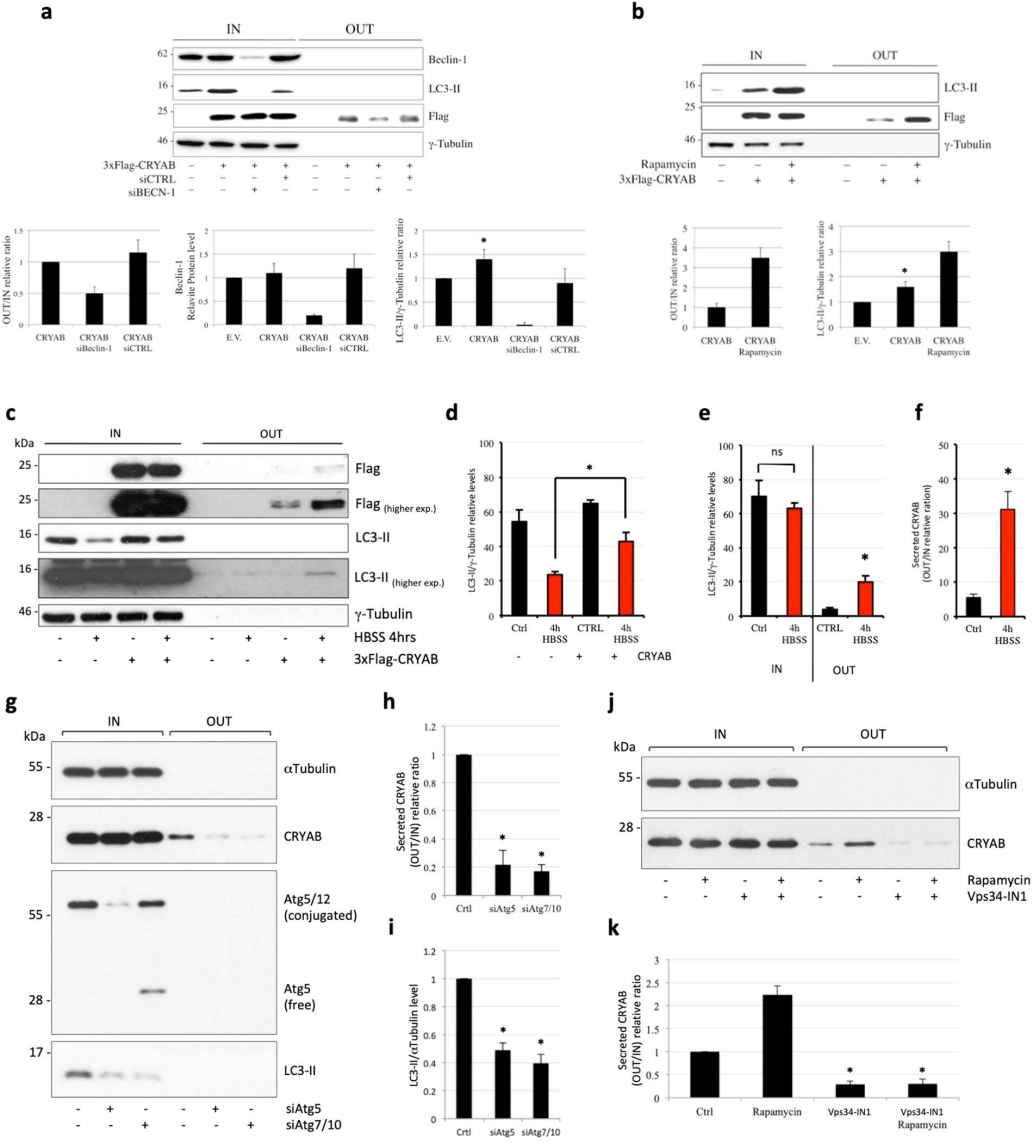
Unconventional secretion of endogenous CRYAB requires autophagic pathway. (**a**–**c)** COS-7 cells were transiently transfected with 3xFlag-CRYAB and 48 hours post-transfection the lysates were separated on SDS-PAGE and the proteins were revealed by using the antibodies indicated on the right of the gels. CRYAB secretion rate was estimated in co-transfected cells with siBECN-1 or siCTRL (**a**), after Rapamycin (**b**) or HBSS treatment (**c)**. (**d–f)** The graph in (**d**) reports the quantitative analysis of LC3-II levels expressed as relative ratio to loading control. The graphs in (**e**,**f**) report the LC3-II and 3xFlag-CRYAB levels expressed as relative ratio between extracellular (OUT) and intracellular (IN) pool. The P value for assessing the effect of autophagy inhibition on CRYAB secretion was determined using Student’s t-test (n = 3; NS = non-significant). (**g–i)** COS-7 cells were transfected for 72 hours with 100 nM of control siRNA or siRNAs against either Atg5 or Atg7/10 to inhibit autophagosome biogenesis. The intracellular and extracellular fractions were collected in the last 6 hours. The graphs in (**h**,**i**) reports the quantitative analysis of endogenous CRYAB levels expressed as relative ratio between extracellular (OUT) and intracellular (IN) pool. The P value for assessing the effect of autophagy inhibition on CRYAB secretion was determined using Student’s t-test (n = 3; NS = non-significant). (**j,k)** COS-7 cells seeded on 6-multiwell were treated for 6 hours with DMSO, rapamycin, the VPS34-IN1 inhibitor or the combination of both (rap/VPS34-IN1). The graph reports the quantitative analysis of endogenous CRYAB levels expressed as relative ratio between extracellular (OUT) and intracellular (IN) pool. The P value for assessing the effect of autophagy modulation on CRYAB secretion was determined using Student’s t-test.

As previously reported, CRYAB is secreted by RPE cells in a Multi-Vesicular Bodies (MVB)-dependent manner^27^. However, a growing body of evidences derived from studies conducted both in yeast and in mammalian cell-based systems support the involvement of autophagic pathway in UPS^11,13,14^. In particular, it has been proposed that proteins can be initially recruited into phagophores, during the early steps of autophagosome formation. The second step consists in the subsequent fusion of these compartments with MVBs, followed by their fusion with plasma membrane that eventually leads to the release of the bodies out of the cells^30^. To investigate whether autophagic pathway might be involved in the unconventional secretion of CRYAB, COS-7 cells were transiently transfected with 3xFlag-CRYAB in the presence or in the absence of the siRNA against Beclin-1, a key regulator of the autophagic pathway activity^31^ (Fig. 1a). As expected, in the presence of the specific siRNA, both the Beclin-1 protein (histogram in the middle) and the phosphatidylethanolamine (PE)-conjugated form of LC3 (LC3-II) levels were drastically reduced, indicating that the autophagic pathway was efficiently inhibited. Interestingly, in this condition the amount of secreted CRYAB was clearly reduced by about 50% (histogram on the left). Conversely, treatment with the known autophagy inducer rapamycin, increased both the level of the LC3-II (Fig. 1b, histogram on the right) and CRYAB secretion, compared to untreated cells (Fig. 1b, histogram on the left).

Finally, in order to assess whether a more physiologic induction of autophagy may influence the unconventional secretion rate of CRYAB, HeLa cells were transiently transfected with 3xFlag-CRYAB and after 24 hours were subjected to full amino acids starvation, by incubating cells with HBSS medium, as previously reported^32^. As reported in Fig. 1c,f, following to 4 hours of treatment with HBSS buffer (+HBSS), the rate of CRYAB secretion was highly increased (about 6-fold), compared to cells kept in full medium (−HBSS). Noteworthy, full amino acids starvation (4 hours HBSS) reduced LC3-II protein levels, likely as a consequence of an increased LC3-II turnover by autophagic flux induction, as previously reported^33–35^, whereas, upon CRYAB over-expression, such a reduction was less pronounced (Fig. 1d). In addition, it was possible to detect a significant, albeit small, proportion of LC3-II (i.e. autophagosomes) in conditioned culture medium obtained from cells grown in full medium conditions (Fig. 1d, higher exposure), with such proportion being increased by HBSS treatment (Fig. 1e,f). In order to further corroborate these observations, we used two different approaches. Firstly, we did perform siRNA-mediated knock-down experiments by depleting either Atg5 or Atg7/10 in combination, which are key autophagy genes whose activity is essential for regulating the early step of autophagosome biogenesis. As expected, we could appreciate the reduction of Atg5 (and of the critical Atg5/12 conjugate) (Fig. 1g, middle panel), as well as a significant reduction in LC3-II levels (Fig. 1g, bottom panel). In both cases, as opposed to control knock-down cells, we observed a significant reduction in the fraction of endogenous CRYAB secreted by COS-7 cells. (Fig. 1g–i).

As a complementary approach, we used Vps34-IN1^36^, a well characterized selective inhibitor of the Class-III PI3Kinase Vps34, which drives PI3P synthesis and is required for the assembly of the phagophore. As expected, we could observe an early-onset and substantial reduction of autophagosome synthesis (about 50%), by using a 1μM concentration of the Vps34-IN1 inhibitor for 6 hours, both in HeLa and COS-7 cells (Fig. S3). In the same experimental setting, we could observe how the VPS34-IN1 treatment was able to reduce the secretion of endogenous CRYAB in COS-7 cells, compared to DMSO-treated cells. More importantly, Vps34-IN1 was also able to effectively antagonize the (rapamycin-induced) increased secretion of CRYAB (Fig. 1j,k). All together, these results strongly suggest the involvement of the autophagic pathway in the unconventional secretion of CRYAB.

### CRYAB over-expression prevents autophagosomes fusion with lysosomes

**Figure 2:**
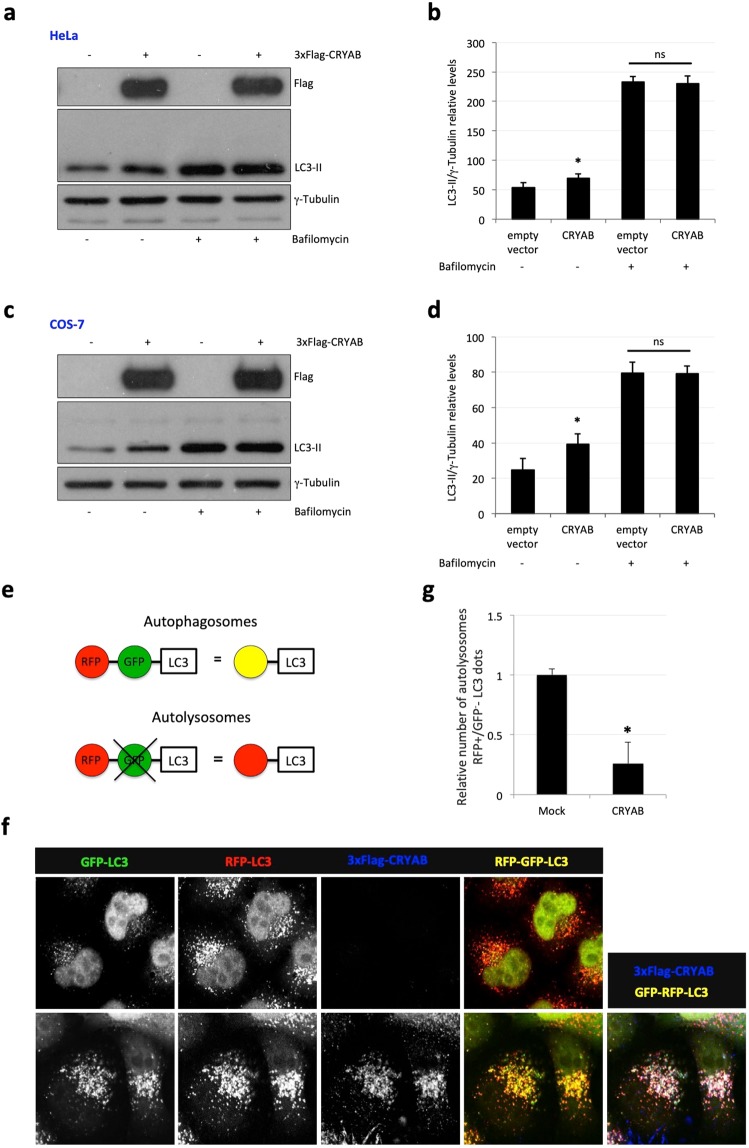
CRYAB over-expression reduces turnover of LC3-II. HeLa **(a)** or COS-7 **(c)** were transiently transfected with 3xFlag-CRYAB and 48 hours post-transfection cells were treated with Bafilomycin A_1_ for 4 hours and lysates were separated on SDS-PAGE. Proteins were revealed by using the antibodies indicated on the right side of the gels. The histograms **(b,d)** indicate LC3-II protein levels (*P value < 0.001; ns = not statistically significant). (**e)** Schematic view of traffic-light assay. (**f**,**g)** HeLa cells, stably expressing RFP-GFP-LC3 protein, were transiently transfected with 3xFlag-CRYAB and after 48 hours were fixed, subjected to the Immunofluorescence and analysed by confocal microscopy. RFP-LC3, GFP-LC3, CRYAB or merge signals were indicated on the top of the panels. Single focal sections are shown. Scale bar: 10 μm.

To test whether CRYAB over-expression might have an effect on autophagosome synthesis, we used two different approaches. First, we measured LC3-II/tubulin relative levels, clamping or not the LC3-II/autophagosome degradation with the lysosomal proton pump inhibitor Bafilomycin A_1_. In either HeLa (Fig. 2a,b) or COS-7 cells (Fig. 2c,d), CRYAB over-expression increased, compared to mock-transfected cells, the steady-state levels of LC3-II, but without causing any further increase in Bafilomycin A_1_-treated cells. Thus, we reasoned that CRYAB over-expression could somehow reduce the LC3-II turnover and degradation^37^.

Then, in order to further elucidate how CRYAB over-expression could influence autophagic flux, we moved to the HeLa cells stably expressing the RFP-GFP-LC3 reporter^38^. Based on the large difference of the pKa value of the two fluorescent proteins, this construct can be used as a probe for autophagosome maturation. At physiological pH (i.e. in newly formed autophagosomes) both fluorescent proteins are stable, leading to emission of red and green fluorescence. Upon acidification (after fusion with the lysosome), the green fluorescence is rapidly lost because of the high pKa of the GFP reporter and only red fluorescence remains (Fig. 2e). Since at the steady state, both LC3 and CRYAB proteins show a largely cytosolic (i.e. non-membrane associated) localization (Fig. S3, left panels), which could have certainly affected the co-localization analysis, cells were firstly subjected to a semi-permeabilization step by digitonin treatment, as previously described^39^. Such procedure allows one to substantially reduce the diffuse background staining deriving from the non-membrane associated fraction of both CRYAB and LC3. Nonetheless, the nuclear as well as the vesicular localization of the two proteins was still preserved (Fig. S3, right panels).

**Figure 3:**
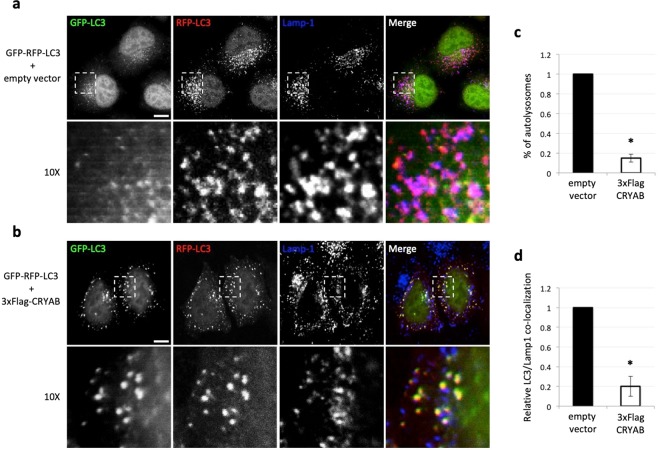
Autophagosomes containing CRYAB do not fuse with lysosomes. (**a,b)** HeLa cells, stably expressing RFP-GFP-LC3 protein, were transiently transfected as indicated on the left of the panels and 48 hours post-transfection were fixed and processed for the immunofluorescence by using a mouse monoclonal anti-Lamp-1 antibody (marker of the late endosome/lysosomal compartment). Single focal sections are shown. Scale bar: 10 μm. (**c)** Quantification of RFP^+^-GFP^−^-LC3 co-localization with late endosomal marker Lamp1. The analysis was performed using the JaCoP plug-in (ImageJ). The co-localization was calculated by using the Mander’s co-localization coefficient. The graph shows the normalized values of coefficients, where the co-localization between RFP^+^-GFP^−^-LC3 and Lamp1 was set to 1. The *p* values were determined by using Student’s t-test (n = 30 from three independent experiments; *P < 0.001). (**d)** The percentage of autolysosomes was calculated measuring the number of RFP^+^-GFP^−^-LC3 positive structures. The graph shows the normalized values relative to the number of RFP^+^-GFP^−^-LC3 structures before and after 3xFlag-CRYAB over-expression. The *p* values were determined as in (**c**).

As shown in Fig. 2g, CRYAB over-expressing cells showed an increased number of autophagosomes (RFP^+^-GFP^+^ structures) and a reduced number of autolysosomes (RFP^+^-GFP^−^ structures, Fig. 2f). Interestingly, we also noticed that CRYAB strongly co-localized with LC3 positive structures (please, see the merge panel in Fig. 2g). These observations are consistent with the effect of CRYAB over-expression on the increased LC3-II protein level (Fig. 2a–d). Such an effect may be due either to the inhibition of lysosomes acidification or fusion of autophagosomes with lysosomes. In order to discriminate between these two possibilities, we analyzed the co-localization of LC3 with the late endo-lysosomal membranes marker Lamp-1 by confocal immunofluorescence microscopy. As shown in Fig. 3, in empty vector transfected cells the RFP^+^-GFP^−^−LC3 autolysosomes showed an extensive co-localization with Lamp-1, indicating that autophagosomes were able to fuse with lysosomes. In contrast, upon CRYAB over-expression, the number of RFP^+^-GFP^−^-LC3 autolysosomes was reduced (consistent with the effect shown in Fig. 2f,g) and, most importantly did not co-localize with Lamp-1. Furthermore, the RFP^+^-GFP^+^-LC3 autophagosomes did co-localize with Lamp-1 to a lesser extent (Fig. 3, please refer to the magnified inserts), compared to the mock transfected cells. These data strongly support that CRYAB over-expression reduces the fusion of autophagosomes with lysosomes.

### Autophagy modulation does not influence the rate of MVB-mediated secretion

**Figure 4:**
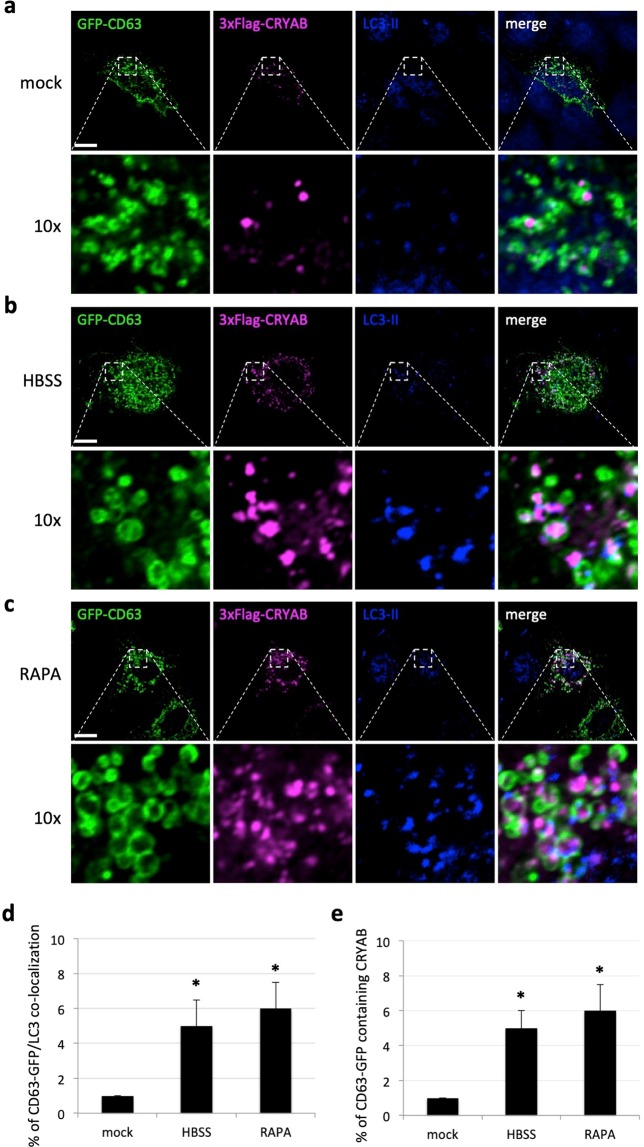
Autophagy modulation does not influence the rate of MVB-mediated secretion. HeLa cells were transiently transfected with the 3xFLAG-CRYAB and GFP-CD63 over-expression constructs as indicated on the left of the panels and 48 hours post-transfection were fixed and processed for immunofluorescence analysis by using a mouse monoclonal anti-LC3 and the mouse monoclonal anti-FLAG antibodies. Single focal sections of mock **(a)**, HBSS **(b)** or rapamycin (RAPA) **(c)** treated cells acquired by confocal microscopy are shown. Scale bar: 10 μm. (**d,e)** Quantification of colocalization between GFP-CD63 and LC3 (**d**) or CRYAB (**e**). The co-localization coefficients were shown as normalized values as in Fig. 3. The *p* values were determined by using Student’s t-test (n = 30 from three independent experiments; *P < 0.001).

In view of the intimate correlation existing between the endo-lysosomal and autophagosomal pathways in mammalian cells, we decided to further characterize the trafficking intersections which have been recently described in the context of unconventional secretion. In particular, we set out to assess whether the modulation of autophagic pathway could provide a functional bridge and contribute to the exosome-dependent secretion of CRYAB, which involves the delivery to and through the multi-vesicular-bodies (MVB) membrane system^27^. To this aim we did perform confocal imaging analysis of 3xFlag-CRYAB in HeLa cells transiently over-expressing GFP-CD63, a well-known marker of MVB bodies^40^. As expected, by looking at the intracellular distribution of CRYAB positive structures (upon digitonin semi-permeabilization), in steady-state conditions we could observe only a partial co-localization between CRYAB and CD63-positive structures Fig. 4a–e), likely because of the transient presence of CRYAB in those structures. Such an observation is in agreement with the previously published literature, linking the exosomal pathway to the unconventional secretion of CRYAB. Furthermore, we showed how upon induction of autophagy by either HBSS or rapamycin (RAPA), this effect was more pronounced (Fig. 4b,c,e). However, we went on to assess the effect of autophagy perturbation on the MVB-mediated secretion and we found some surprising results. Indeed, by measuring the amount of GFP-CD63 secreted in Hela cells depleted of either Atg5 or Atg7/10 to inhibit autophagosome biogenesis, we did not observe any reduction, compared to control cells (Fig. S5). On the same line, the chemical inhibition (by means of the Vps34-IN1) or activation (by rapamycin) of the pathway did not show any significant change in the amount of CD63 secreted in the extracellular compartment (Fig. S6).

Hence, we can confirm the existence of a functional intersection between the autophagosomal and MVB-membrane systems, which is corroborated by our confocal imaging analysis (Fig. 4a–c). Nevertheless, at least in our experimental set-up, the modulation of the autophagic pathway, albeit being capable of varying the amount of (both endogenous and exogenous) secreted CRYAB (please, refer to Fig. 1g–m), does not have any overt effect on the exosomal secretion, as assessed by measuring the fraction of GFP-CD63 positive structures secreted out of the cells (Figs S5 and S6).

### Serine 59 negatively regulates CRYAB secretion

**Figure 5:**
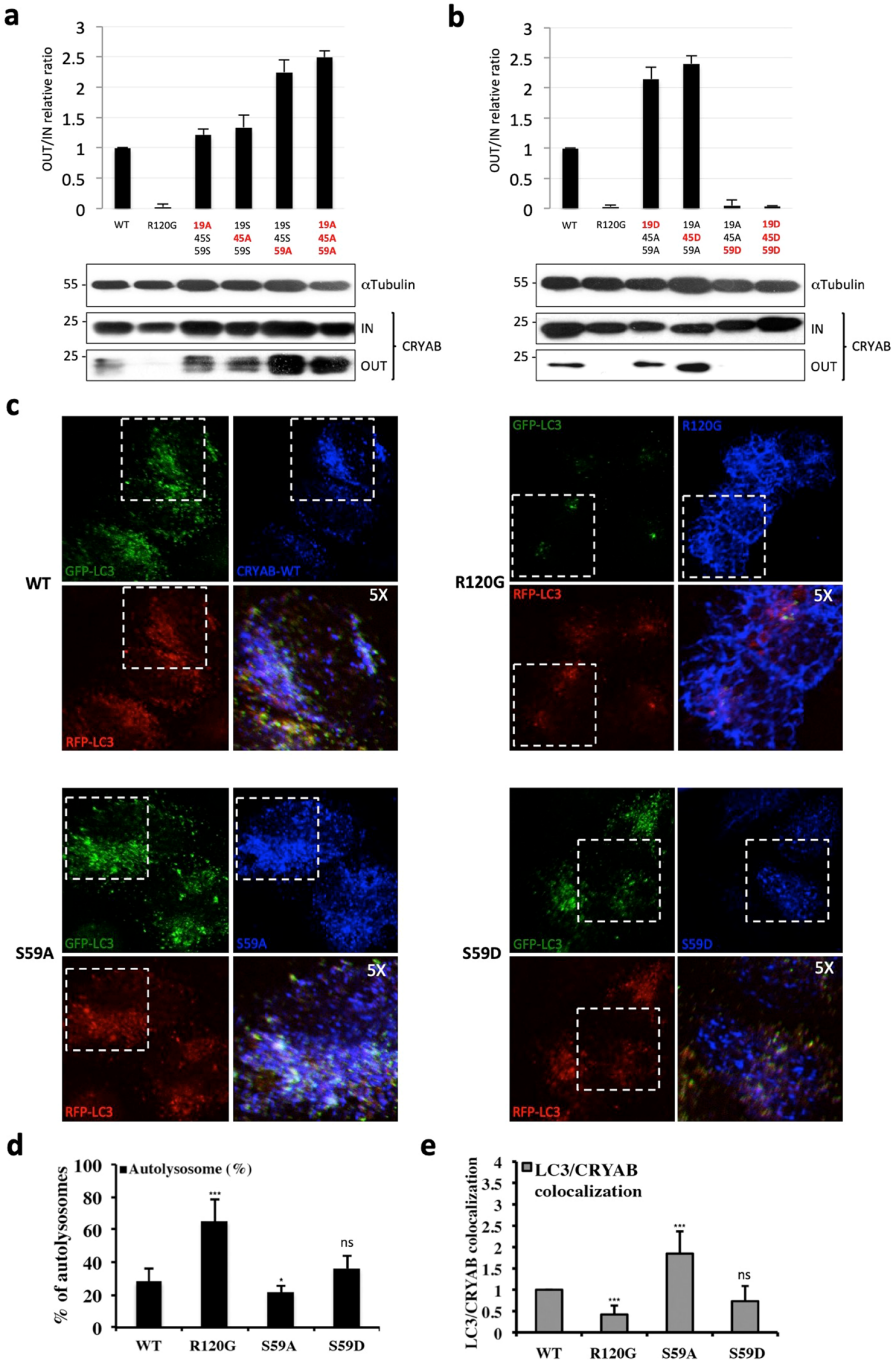
Serine 59 controls CRYAB secretion. (**a,b)** Equal amount of both cell lysates and extracellular media of COS-7 cells transiently transfected with wyld type 3xFlag-CRYAB or its not-phosphorylatable/pseudo-phosphorylated mutants were analysed by SDS-PAGE and CRYAB proteins were revealed by using a mouse monoclonal anti-Flag antibody. (**c,d)** HeLa cells, stably expressing RFP-GFP-LC3 protein, were transiently transfected with the indicated constructs and after 40 hours were fixed and subjected to the immunofluorescence by using a mouse monoclonal anti-Flag antibody to recognize CRYAB proteins while LC3 proteins were detected by auto-fluorescence for the presence of GFP- and RFP-tagged proteins. (**e)** Quantification of RFP-GFP-LC3 co-localization with wild type and mutant forms of CRYAB. The analysis was performed using the JaCoP plug-in (ImageJ). The co-localization between CRYAB and LC3-positive structures were calculated by using the Mander’s co-localization coefficient. The graph shows the normalized values of coefficients, where the co-localization between LC3 and wild type CRYAB was set to 1. The *p* values for assessing the RFP-GFP-LC3 and CRYAB co-localization were determined by using Student’s t-test (n = 10; ***P < 0.001; **P < 0.01; *P < 0.05; NS: not-significant). A single focal section is shown. Scale bar: 10 μm.

CRYAB bears three serine residues that can undergo phosphorylation, namely: Ser19, for which the kinase is not known, Ser45 and Ser59, which can be phosphorylated by p44/42 mitogen-activated protein kinase and MAP kinase-activated protein kinase-2 (p38), respectively^41,42^. As previously shown, the phosphorylation switch on these sites do confer a different subcellular localization in cultured hippocampal neurons^43^ and influence its chaperone-like activity^19,44,45^. In order to investigate whether CRYAB phosphorylation could also influence its secretion rate, we generated by site-direct mutagenesis (as detailed in the Materials and Methods section) the three single not-phosphorylatable mutants S19A, S45A, S59A, as well as the triple mutant S19A/S45A/S59A (3A). On the other hand, in order to avoid any possible biological output generated by cellular signaling events affecting at the same time multiple phosphorylation sites, the three single pseudo-phosphorylated mutants (S19D, S45D and S59D), as well as the triple mutant S19D/S45D/S59D (3D), were generated (Fig. S7) on the 3A background. As shown in Fig. 5a, abolishing phosphorylation either in position 19 or 45 showed a minor effect on CRYAB secretion, whereas the S59A (as well as the 3A) mutant showed a hugely increased level of secretion (about 2-fold), compared to the wild-type CRYAB-transfected cells. Interestingly, neither the S19D nor the S45D substitutions were able to decrease the secretion of the non-phosphorylatable 3A mutant, whereas either S59D or 3D mutant almost abolished CRYAB secretion, compared to wild-type CRYAB (Fig. 5b).

Then, in order to investigate whether the observed difference in secretion might be due to a different recruitment of CRYAB into autophagosomes, HeLa cells stably expressing RFP-GFP-LC3 were transiently transfected with either the wild-type 3xFlag-CRYAB or its not-phosphorylatable/pseudo-phosphorylated mutants. Interestingly, the not-phosphorylatable S59A mutant showed a higher co-localization with autophagosomal structures (Fig. 5c,e), whereas the pseudo-phosphorylated S59D mutant showed, compared to the wild-type CRYAB, an overall similar association and co-distribution with LC3-postive structures (Fig. 5c,e). Likewise, the same approach was applied in COS-7 cells co-transfected with GFP-LC3 in combination with either wild-type or mutant CRYAB isoforms and the co-localization was assessed and expressed as the number of GFP-LC3 dots positive for CRYAB (Fig. S8a,b).

Moreover, we found that the disease-associated CRYAB-R120G mutant, which is defective in its chaperone activity^18,46^, was not secreted by COS-7 cells (Fig. 5a,b). In consideration of its spontaneous tendency to form insoluble cytosolic aggregates, its over-expression was found to cause a marked increase of autolysosomes (Fig. 5d) and, as one would expect for an autophagic substrate, a reduced co-localization with the autophagosome-positive structures (Figs 5e and S9a). This result prompted us to understand whether the R120G mutant was not capable to divert autophagosomes toward the exocytic pathway because it is a substrate of autophagy-dependent degradation pathway and, as such, not secreted by the cell. In order to address such a possibility, we did perform substrate clearance experiments in HeLa cells over-expressing the 3xFlag-CRYAB-R120G mutant, by inducing (or blocking) autophagy with trehalose or bafilomycin A_1_, respectively (Fig. S9b,c). Notably, in these experiments we also monitored the clearance of the Parkinson’s disease-associated A53T α-synuclein mutant, a known autophagy substrate, whose intracellular levels mirror the activity of the autophagic pathway^47^. Differently from the expression levels of A53T α-synuclein, neither trehalose nor bafilomycin A_1_ were able to significantly change the levels of CRYAB-R120G mutant (Fig. S9b,c). Therefore, the turn-over of the CRYAB-R120G mutant protein does not rely on the autophagic pathway.

All together, these results indicate that CRYAB secretion is negatively regulated by the phosphorylation state of its Serine 59 residue, which hampers the recruitment of the protein into autophagosomal structures.

## Discussion

To our knowledge, this is the first systematic study in mammalian cells that links the phosphorylation-dependent unconventional secretion of α-Crystallin B (CRYAB), a member of the small heat-shock proteins (sHsp) superfamily with chaperone activity^48^, to the activity of the autophagic pathway. More specifically, the characterization of the mechanisms regulating the secretion of α-Crystallin B has led us to three key findings: i. CRYAB is unconventionally secreted in autophagy-dependent manner; ii. when recruited, CRYAB is capable to highjack and divert autophagosomes from the canonical route (leading to the lysosomal compartment) toward the exocytic pathway; iii. the phosphorylation state of the CRYAB serine 59 residue can regulate its own secretion by influencing the recruitment to the autophagosomal structures.

### The autophagic pathway contributes to CRYAB unconventional secretion

We report evidences that CRYAB can be actively secreted in exocytic vesicles. Moreover, the rate of CRYAB secretion can be maintained irrespectively of the activity of the canonical secretory pathway (brefeldin-A treatment, Fig. S1). In mammalian cells, autophagy occurs under basal conditions and can be induced by certain environmental stresses, such as nutrient deprivation, infections, oxidative stress. In starvation conditions, autophagy is induced and increases the availability of nutrients by promoting their release from macromolecules that are targeted for degradation^49^. Although autophagy has been considered in the past as a rather non-selective degradative process, several cargo-specific autophagic processes have been more recently described^49^. In addition, autophagy intersects and share many molecular players with multiple steps of both the endocytic and exocytic pathways^50^. For instance, autophagy has been shown to modulate both constitutive and regulated secretion of some immuno-modulatory cytokines^51^. Interestingly, autophagy can play a role also in the unconventional trafficking of the cystic fibrosis transmembrane conductance regulator (CFTR, the protein mutated in cystic fibrosis) from the ER to the plasma membrane, bypassing the Golgi^52^. Finally, a subset of leaderless proteins can use unconventional secretion processes that rely on autophagic intermediates, structures or vesicular transport carriers, in order to be exported outside the cell^14^. According to our data, CRYAB might be included into the latter category. Indeed, by means of multiple siRNA-based knock-down experiments (Fig. 1g–i), we demonstrated that the basal autophagy contributes to guarantee CRYAB secretion in steady-state conditions. Conversely, by using two different experimental approaches (i.e. induction of autophagy with rapamycin or nutrients starvation with HBSS), we showed that the activation of autophagy can substantially promote CRYAB secretion in our cell-based systems. Finally, by chemically inhibiting the early step of autophagosome biogenesis, we show that autophagy is required for the unconventional secretion of endogenous CRYAB, both at steady-state and in stimulated conditions (Fig. 1j,k).

### CRYAB can divert autophagosomes toward the extracellular compartment

In view of our first line of evidences (Fig. 1), it would have been reasonable to hypothesize that CRYAB over-expression might induce *per se* autophagosome formation. Indeed, we observed a consistent increase in both the LC3-II levels and number of autophagosomes in CRYAB over-expressing cells (Fig. 2). Moreover, most of the autophagosomes resulted positive for CRYAB. An increase in levels of LC3-II can be consequent to either an increased autophagosome formation or a block in autophagosome maturation or degradation. Furthermore, the latter possibility could be explained by a block in autophagosome-lysosome fusion, a defect in lysosomal degradation (which would inevitably reduce the turn-over of autophagosomes), or both. Surprisingly, we found a reduced co-localization between autophagosomes (mostly CRYAB-positive) and the lysosomal compartment, as assessed by endogenous Lamp-1 staining, suggesting that CRYAB delays the delivery (or inhibits the fusion) of autophagosomes with lysosomes (Fig. 3). Nonetheless, we reasoned that the only mild, albeit consistent, increase in the amount of LC3-II levels observed in CRYAB over-expressing cells would not be compatible with the drastically detrimental effects on LC3-II/autophagosomes turn-over one would expect from a block in the later state of autophagy, as it has been previously reported^38,53^.

Such an interesting observation, combined with the active role exerted by autophagy in regulating CRYAB secretion, would be rather compatible with a hypothetical model whereby CRYAB-positive autophagosomes are diverted from the classical route leading to the lysosomal compartment and re-directed toward to the exocytic pathway/plasma membrane, in order to facilitate the secretion of their content. In agreement with these observations, we showed how a substantial portion of the intracellular pool of CRYAB does indeed co-localize with the MVB-marker CD63 (Fig. 4), a phenomenon which is even more pronounced upon autophagy stimulation. Hence, it is conceivable to speculate that a specific subset of trafficking autophagosomes being diverted from the lysosomal degradative system toward secretion routs might well be reaching the MVB-exocytic membrane system. Moreover, this scenario would not be in contradiction with a previous study, reporting that CRYAB is apically secreted by polarized epithelial RPE cells in a multi-vesicular bodies (MVB)-dependent manner^27^. Collectively, our data confirm the existence of a functional intersection between the autophagosomal and MVB-membrane systems. However, the modulation of the autophagic pathway, albeit being capable of varying the amount of secreted CRYAB does not have any overt effect on the MVB-mediated unconventional exosomal secretion. Further experiments will be required in order to shed light and address these interesting issues regarding the interplay between the autophagic and MVB-mediated exocytic pathways.

### Impact of serine 59 phosphorylation on CRYAB secretion

In mammalian cells, heat-shock proteins are mainly localized in the cytosol. Phosphorylation appears to be one of the critical modulators of chaperone functions of small heat shock proteins. However, the role of phosphorylation in regulating CRYAB activity is still not completely understood. In recent years, several studies have investigated the structural and functional consequences of a phosphorylation-mimicking mutation in α-Crystallin B. For instance, the phosphorylation at three serine residues (Ser19, Ser45 and Ser59) represents a major post-translational modification that occurs to alpha B-crystallin in response to stress or during mitosis^42,44^. Furthermore, it has been recently suggested that phosphorylation of α-Crystallin B can account for changes in stability, homo- and hetero-oligomerization properties and reduction of its chaperone-like activity^19,45^. Therefore, we aimed at investigating whether change in phosphorylation state could also influence CRYAB secretion rate. By means of site-directed mutagenesis, we identified the serine 59 as the critical residue in regulating the recruitment into autophagosomal structures and, as a consequence, the secretion rate of CRYAB (Fig. 5). Therefore, it is tempting to hypothesize the existence of a selective, phosphorylation-based mechanism that allows, when necessary, the autophagy-dependent unconventional transport of a specific subset of proteins (including CRYAB), which have to be secreted out of the cell. Notably, and according to some of our data, this scenario could apply also to physiologically relevant conditions, such as in response to nutrient starvation. For instance, it has been reported that in response to nutrient starvation, members of the p38 mitogen-activated protein kinase (MAPK) signaling pathway MAPKAPK2 (MK2) and MAPKAPK3 (MK3) are crucial stress-responsive kinases that promote autophagy through Beclin-1 S90 phosphorylation^54^. Interestingly, the same kinase family members are responsible for the S59 CRYAB phosphorylation^42^, thereby strongly suggesting that these two events are very likely functionally correlated. Further experiments will be required to better address such an interesting possibility.

### Potential implications of CRYAB secretion in the context of disease-associated mutant proteins turn-over

Finally, our findings could be potentially relevant for a number of disease models. For instance, anti-apoptotic and neuroprotective functions have been proposed for the α-Crystallin B (CRYAB) in the development of multiple sclerosis (MS). In particular, in early-phase active MS lesions, extracellular α-Crystallin B is available for functional presentation to T-cells during the inflammatory demyelination process^55–57^. In this context, CRYAB represents a potent negative regulator acting as a brake on several pro-inflammatory pathways in both the immune system and central nervous system. In the context of multiple sclerosis, the immune response against CRYAB exacerbate inflammation and demyelination *in vivo* and is overall detrimental for the pathological outcome^26^. Thus, administration of recombinant CRYAB has been proposed for therapy of ongoing disease. As a further example, α-Crystallin B has been found, along with α-synuclein, as a major component of Lewy bodies that are characteristic of Parkinson’s disease (PD). CRYAB is a potent inhibitor of α-synuclein amyloid fibrils formation^58,59^. The two mutant forms (A30P and A53T) of α-synuclein, that are causative of familial, early-onset forms of PD, are autophagy substrates and pharmacological up-regulation of autophagy has been shown to enhance the clearance of these mutant proteins, resulting beneficial in the progression of several neurodegenerative disease models^49^. Therefore, the novel mechanism we describe here, by which α-Crystallin B (and its interacting proteins) is recruited in autophagosomes, might be relevant and deserves further investigations, as it could represent an additional “back-up” system by which cells are able to cope with and alleviate the accumulation of intracellular mutant, aggregate-prone or misfolded proteins. As such, this could ultimately limit the pathogenesis and the onset of misfolding diseases, and in perspective, represent a potential target to develop novel therapeutic intervention.

## Materials and Methods

### Reagents

All of the culture reagents were obtained from Sigma-Aldrich (Milan, Italy). The solid chemical and liquid reagents were obtained from E. Merck (Darmstadt, Germany), Farmitalia Carlo Erba (Milan, Italy), Serva Feinbiochemica (Heidelberg, Germany), Delchimica (Naples, Italy) and BDH (Poole, United Kingdom). Protein A-Sepharose CL-4B and the enhanced chemiluminescence reagents were from Roche (Milan, Italy). Brefeldin-A (BFA), digitonin, rapamycin, bafilomycin A_1_, trehalose and cycloheximide were purchased from Sigma-Aldrich, Milan, Italy. The Vps34-IN1 inhibitor (CAS № 1383716-33-3) was purchased from Cayman Chemicals (Ann Arbor, Michigan-USA).

### Antibodies

The following antibodies were used: Peroxidase conjugated anti-mouse and anti-rabbit IgG (Sigma-Aldrich, Milan, Italy); Texas-Red-conjugated anti-mouse and anti-rabbit IgG, FITC-conjugated goat anti-mouse and anti-rabbit IgG, Cy5-conjugated goat anti-mouse and anti-rabbit IgG (Jackson ImmunoResearch Laboratories, West Grove, PA); Mouse monoclonal anti-FLAG antibody (Sigma-Aldrich, Milan, Italy); Mouse monoclonal anti-CRYAB antibody (Enzo Life Sciences, USA). Mouse monoclonal anti-α-tubulin antibody (Sigma-Aldrich, Milan, Italy); Rabbit polyclonal anti-LC3 antibody (Novus Biologicals); Mouse monoclonal anti-LC3 antibody (NanoTools); Mouse monoclonal anti-CD7107a (Lamp-1) antibody (Biolegend, San Diego, CA); Rabbit polyclonal anti-BECN1 (Santa Cruz Biotechnology); rabbit polyclonal anti-GFP antibody (AbCam); rabbit polyclonal anti-Atg5 antibody (AbCam). Rabbit polyclonal anti-GM130 antibody was described previously^60^.

### Constructs, siRNA reagents, cDNA cloning and plasmid construction

The pCMV6-XL5 expression vector for human CRYAB protein (ID NM_001885.1) was obtained from I.M.A.G.E. Consortium. To generate the 3xFlag-CRYAB construct, the cDNA coding for CRYAB was amplified by PCR from the pCMV6-XL5 plasmid using the following primers (containing HindIII/XbaI flanking restriction sites) and cloned into the p3xFlag-CMV-7.1 expression vector: Fw (HindIII): 5′-AAGCTTATGGACATCGCCATCCACCACCC-3′; Rv (XbaI): 5′-TCTAGACTATTTCTTGGGGGCTGCGG-3′. To obtain the 3xFlag-CRYAB-R120G construct, in which the R_120_ was substituted with a glycine residue (G), the construct 3xFlag-CRYAB was used as a template and site-direct mutagenesis was performed according to the manufacturer instructions and as previously reported^61,62^ by using the following primers: Fw: 5′-CTCCAGGGAGTTCCACGGGAAATACCGGATCCCAG-3′; Rv: 5′-GTGGAACTCCCTGGAGATGAAACC-3′. To generate the CRYAB mutant constructs carrying the not-phosphorylatable sites (S19A, S45A, S59A and S19/45/59A), the following primers were used: Fw: CTTCTTTCCTTTCCACGCCCCCAGCCGCCTCTTTG; Rv: AAGAAGGGGCGGCGGATCCAG for S19A; Fw: CGTCTACTTCCCTGGCTCCCTTCTACCTTCGG; Rv: CAGGGAAGTAGACGTCGGGAAAAG for S45A; Fw: CTTCCTGCGGGCACCCGCCTGGTTTGACACTGGAC; Rv: GGGTGCCCGCAGGAAGGAGGG for S59A. The combination of them was used to obtain the triple mutant 3xFlag-CRYAB-S19/45/59A.

To obtain the pseudo-phosphorylated mutants of CRYAB (S19D, S45D, S59D and S19/45/59D) the 3xFlag-CRYAB-S19/45/59A construct was used as a template and site-direct mutagenesis was performed using the following primers: Fw: CTTCTTTCCTTTCCACGACCCCAGCCGCCTCTTTG; Rv: AAGAAGGGGCGGCGGATCCAG for S19D; Fw: CGTCTACTTCCCTGGACCCCTTCTACCTTCGG; Rv: CAGGGAAGTAGACGTCGGGAAAAG for S45D; Fw: CTTCCTGCGGGCACCCGACTGGTTTGACACTGGAC; Rv: GGGTGCCCGCAGGAAGGAGGG for S59D. The combination of them was used to obtain the triple mutant 3xFlag-CRYAB-S19/45/59D construct.

For the knock-down experiments, Lipofectamine 2000 Transfection Reagent was used according to the manufacturer’s instructions (Thermofisher Scientific, Cat. No.: 11668019), in combination with the following siRNAs: ON-TARGETplus Non-targeting Pool (Cat. No.: D-001810-10-50), SMARTpool: ON-TARGETplus ATG5 (Cat. No.: L-004374-00-0010), SMARTpool: ON-TARGETplus ATG7 (Cat. No.: L-020112-00-0010), SMARTpool: ON-TARGETplus ATG10 (Cat. No.: L-019426-01-0010) (Dharmacon, Horizon Discoveries).

The plasmid encoding GFP-CD63 was obtained from Paul Luzio’s lab (Addgene plasmid #62964). The plasmids encoding empty GFP (peGFP-C1, Clontech) and GFP-A53T alpha-synuclein (Addgene plasmid #40823) were obtained from David Rubinsztein’s lab. The peGFP-LC3 expression vector and siRNA targeting Beclin-1 were kindly provided by Maria Fiammetta Romano^63^.

### Cell culture and transfection experiments

HeLa and COS-7 cells were routinely grown at 37 °C in Dulbecco’s modified essential medium (DMEM), containing 10% foetal bovine serum (FBS), 100 U/ml Penicillin/Streptomycin, 2 mM l-Glutamine (l-Gln). HeLa cells stably expressing the RFP-GFP-LC3 reporter^39^ were grown in the same medium, supplemented with 600 μg/ml G418 (Gibco). Cells were transfected by using FuGene 6.0 (Roche, Milan, Italy) according to the manufacturer’s instructions. For the full amino acid starvation experiments, cells were incubated for 4 hours in Hank’s Balanced Salt Solution (HBSS, Gibco-Brl).

### Immunofluorescence and confocal microscopy

Indirect immunofluorescence was performed as previously described^64–66^. Single confocal images were acquired at 63x magnification on a LSM510 Meta (Carl Zeiss, Jena, Germany). For each co-transfection, 30 cells were considered for quantification. In traffic-light experiment the number of RFP^+^-GFP^−^-LC3 dots were measured as difference between RFP^+^-GFP^−^-LC3 and RFP^+^-GFP^+^-LC3 dots by using *ImageJ Biophotonics software*. For the quantification of GFP-LC3 co-localization with CRYAB phosphorylation mutants, the number of red dots (CRYAB) co-localizing with green dots (GFP-LC3) was measured by using *ImageJ Biophotonics software*. The results are given as mean ± s.d.

### Semi-permeabilization assay by digitonin treatment

In order to better visualized the colocalization between GFP-LC3 and 3xFlag-CRYAB in COS-7, after 48 hrs from transfection cells were semi-permeabilized, as previously described^39^. Briefly, cells were transferred to ice and washed immediately with ice-cold KHM buffer (110 mM KOAc, 20 mM Hepes, pH 7.2, 2 mM MgOAc). Cells were digitonin permeabilized at 40 μg/ml in KHM buffer for 3 min on ice, fixed in 3,7% formaldehyde in PBS for 30 min at room temperature after wash with ice-cold KHM buffer and subjected to indirect immunofluorescence as described above. Single confocal images were acquired at 63x magnification on a LSM510 Meta (Carl Zeiss, Jena, Germany).

### Preparation of cell extracts, SDS-PAGE and Western Blot analysis

Preparation of cell extracts, SDS-PAGE and Western blot analysis were performed as previously detailed^67,68^.

## Supplementary information

Supplemenrtary informations
