# Supplementary material for: Unconventional secretion of α-Crystallin B requires the Autophagic pathway and is controlled by phosphorylation of its serine 59 residue
Source: Sci Rep. 2019 Nov 15;9:16892. doi: 10.1038/s41598-019-53226-x (PMC6858465; doi:10.1038/s41598-019-53226-x)

SUPPLEMENTARY INFORMATION FILE

Unconventional secretion of α-Crystallin B requires the Autophagic pathway and is controlled by phosphorylation of its serine 59 residue

D'Agostino M.[1#], Scerra G.[1], Cannata Serio M. [2,3], Caporaso MG. [1], Bonatti S.[1], Renna M.[1]*

[1]Department of Molecular Medicine and Medical Biotechnologies, University of Naples Federico II, Italy
[2]Laboratory of Epithelial Biology and Disease, Imagine Institute, Paris, France
[3]Université Paris Descartes-Sorbonne Paris Cité, Imagine Institute, Paris, France
*Corresponding author: correspondence to maurizio.renna@unina.it
[#]Co-Corresponding author: correspondence to massimo.dagostino@unina.it

SUPPLEMENTARY FIGURE LEGENDS

Fig. S1. Characterization of the expression profile of endogenous CRYAB in monkey kidney fibroblast COS-7 cell line.

a) Equal amounts of lysates from mock COS-7 cells or transiently transfected one with the tagged (3xFlag-CRYAB) and non-tagged (h-CRYAB) forms of CRYAB were analyzed by SDS-PAGE. The endogenous and exogenous forms were revealed by using a specific antibody against CRYAB or its 3xFlag tag, respectively. **b)** COS-7 cells were fixed and subjected to immunofluorescence by using a mouse monoclonal anti-CRYAB antibody while the nuclei were stained by using DAPI. A single focal section is shown. Scale bar: 10μm.

Fig. S2. Endogenous CRYAB is secreted by unconventional pathway from COS-7 cells.

a) Equal amount of each lysates (IN) and culture media (OUT) of mock (upper row) or transiently transfected COS-7 cells with 3xFlag-CRYAB (lower row) were separated on SDS-PAGE and CRYAB proteins were revealed by using a specific antibody against the endogenous form (CRYAB Endo) or a mouse monoclonal anti-Flag antibody to detect the exogenous form (3xFlag-CRYAB). The histogram on the right indicates the OUT/IN ratio of both endogenous or exogenous form of CRYAB, respectively. **b)** CRYAB secretion rate were estimated in COS-7 cells transiently transfected as in A in the presence or absence of Brefeldin A treatment (BFA). The immunofluorescence images on the right show the effect of BFA treatment on Golgi tubulation/fragmentation revealed by using a rabbit polyclonal anti-GM130 antibody (marker of Golgi complex) Scale bar: 10μm. **c)** Exosomes isolation from culture media of COS-7 cells transiently transfected with 3xFlag-CRYAB before or after Triton X-100 treatment. CRYAB proteins were revealed by using a mouse monoclonal anti-Flag antibody. The graph in a reports the quantitative analysis of CRYAB OUT/IN relative ratio from independent experiments performed in triplicate (n=3; *P<0.05).

Fig. S3. Preliminary validation of the Vps34 chemical inhibition as a tool to down-regulate autophagy.

a) Chemical structure of the Class-III PI3K inhibitor selective inhibitor Vps34-IN1 (Cayman Chemicals). **b-e)** HeLa (b-c) or COS-7 cells (d-e) were transiently transfected with 1 μg of the GFP-CD63 expression vector for 24 hours. In the last 6 hours, cells were treated with either DMSO or 1 μM of Vps34-IN1 inhibitor. Furthermore, for the assessment of the autophagic flux by LC3-II levels, a saturating concentration (400 nM) of Bafilomycin A_1 was added to the cells in the last 4 hours before harvesting. The graphs in c and e report the quantitative analysis of LC3-II relative to tubulin from independent experiments performed in triplicate (n=3; $^{**}P<0.01$, $^{***}P<0.001$).

Fig. S4. Digitonin-based semi-permeabilization procedure for the analysis of membrane-associated pool of CRYAB/LC3-II.

COS-7 cells transiently transfected with the indicated constructs on the left were subjected to the immunofluorescence before (- Digitonin) and after (+ Digitonin) semi-permeabilization procedure. LC3 proteins were detected by autofluorescence for the presence of GFP fluorescence protein fused at the N-terminus of LC3, whereas CRYAB proteins were detected by using a mouse monoclonal anti-FLAG antibody. A single focal section is shown. Scale bar: 10μm.

Fig. S5. Autophagy inhibition does not influence the rate of MVB-mediated secretion.

a, b) HeLa cells were transfected for 72 hours with 100 nM of control siRNA or siRNAs against either Atg5 or Atg7/10 to inhibit autophagosome biogenesis. **c, d)** In the last 24 hours before collection of the intracellular and extracellular fractions, cells were re-transfected with the same siRNA mix plus 1 μg of the GFP-CD63 expression vector. The graph in d reports the quantitative analysis of GFP-CD63 levels expressed as relative ratio between extracellular (OUT) and intracellular (IN) pool. The P value for assessing the effect of autophagy inhibition on GFP-CD63 secretion was determined using Student's t-test (n=3; ns=non-significant).

Fig. S6. Chemical modulation of the autophagic pathway does not influence the MVB-mediated exosomal secretion.

a-d) HeLa cells were transiently transfected with 1 μg of the GFP-CD63 expression vector for 24 hours. In the last 6 hours before collection, cells were treated with DMSO, 1 μM of Vps34-IN1 inhibitor or 200 nM Rapamycin to either down-regulate (a-b) or up-regulate (c-d) the autophagic pathway, respectively. The graphs in B and D report the quantitative analysis of GFP-CD63 levels expressed as relative ratio between extracellular (OUT) and intracellular (IN) pool. The P value for assessing the effect of autophagy inhibition/induction on GFP-CD63 secretion was determined using Student's t-test (n=3; ns=non-significant).

Fig. S7. Schematic view of phosphorylation mutants of CRYAB.

Mutagenesis was performed as detailed in the materials and methods section.

Fig. S8. Serine 59 influences the recruitment of CRYAB to the autophagosome compartment in COS-7 cells.

a) COS-7 cells transiently transfected with the constructs indicated in the panels were fixed after semi-permeabilization and subjected to the immunofluorescence as described in Fig. S4. **b)** Percentage of co-localization between GFP-LC3 positive-structures and non-phosphorylatable/pseudo-phosphorylated mutants of CRYAB expressed as the relative number of GFP-LC3 dots positive for CRYAB respect to the total number of GFP-LC3 dots present into the same cells. A single focal section is shown. Scale bar: 10μm.

Fig. S9. CRYAB-R120G mutant is not an autophagy substrate.

a) COS-7 cells transiently transfected with the 3xFlag-CRYAB-R120G mutant constructs indicated in the panels were fixed after semi-permeabilization and subjected to the immunofluorescence as described in Fig. S4. Percentage of co-localization between GFP-LC3 positive-structures and CRYAB R120G mutant was reported as the relative number of GFP-LC3 dots positive for CRYAB respect to the total number of GFP-LC3 dots present into the same cells. A single focal section is

shown. Scale bar: 10μm. **b, c)** HeLa cells were transiently transfected with 1 μg of the 3xFlag-CRYAB-R120G, an empty GFP or the GFP/A53T α-synuclein expression vectors. 24 hours after transfection, cells were treated for additional 24 hours with DMSO and either 100 mM Trehalose or 100 nM Bafilomycin A_1 to induce or block the autophagic pathway, respectively. In this setup, the levels of the A53T α-synuclein was used to monitor the clearance of a known autophagy substrate. The graph in c reports the quantitative analysis of GFP-A53T α-synuclein (normalized to GFP) and of CRYAB-R120G relative levels. White arrows indicate the 3xFlag-CRYAB-R120G/GFP-LC3 co-localization structures. The P values for assessing the effect of autophagy inhibition/induction on A53T α-synuclein R120G secretion were determined using Student's t-test (n=3; ns=non-significant, *P<0.05, **P<0.01).

Fig. S1

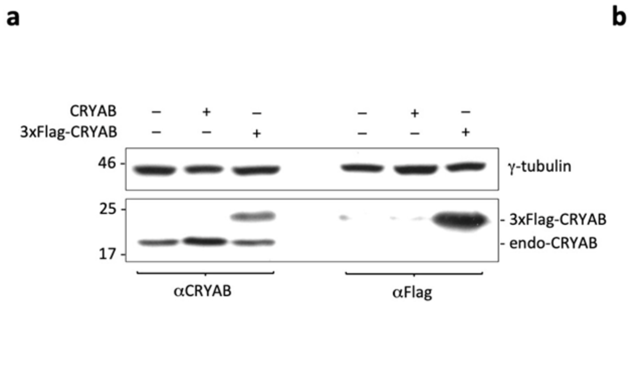

Fig S2

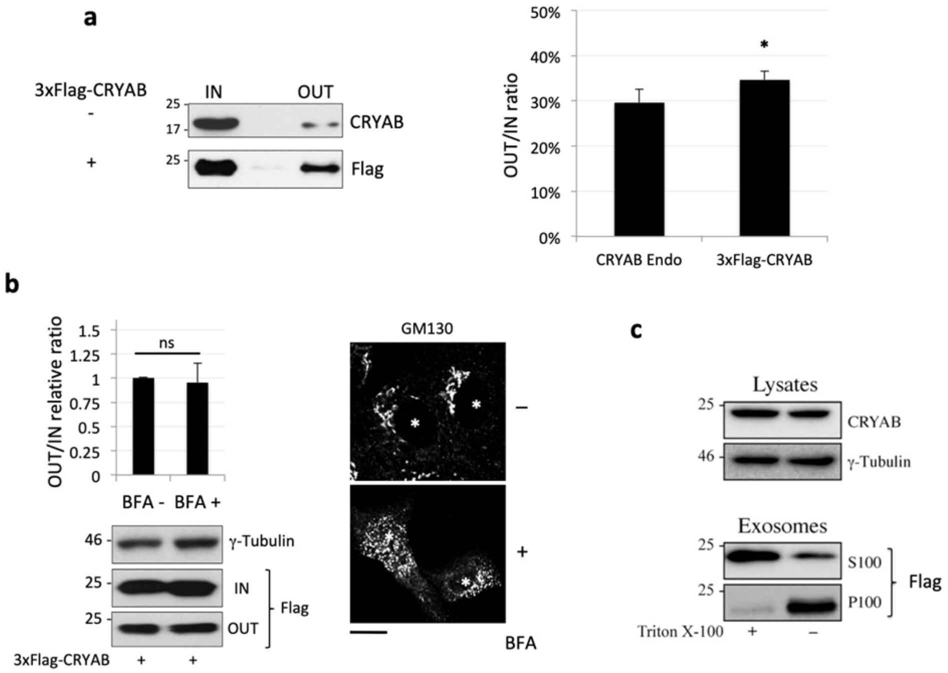
a
3xFlag-CRYAB
-
+
IN
OUT
25
17
25
CRYAB
Flag
50%
40%
30%
20%
10%
0%
OUT/IN ratio
*
CRYAB Endo
3xFlag-CRYAB
b
OUT/IN relative ratio
1.5
1.25
1
0.75
0.5
0.25
0
ns
BFA -
BFA +
46
25
25
γ-Tubulin
IN
OUT
Flag
3xFlag-CRYAB
+
+
GM130
–
+
BFA
c
Lysates
25
46
CRYAB
γ-Tubulin
Exosomes
25
25
S100
P100
Flag
Triton X-100
+
–

Fig. S3

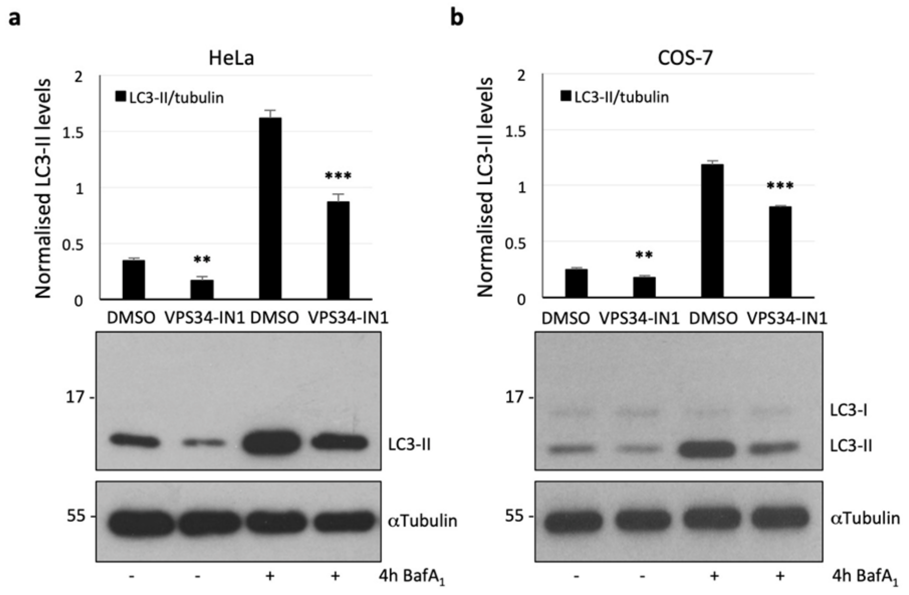
a
HeLa
Normalised LC3-II levels
LC3-II/tubulin
2
1.5
1
0.5
0
**
***
DMSO
VPS34-IN1
DMSO
VPS34-IN1
17
LC3-II
55
αTubulin
-
-
+
+
4h BafA1
b
COS-7
Normalised LC3-II levels
LC3-II/tubulin
2
1.5
1
0.5
0
**
***
DMSO
VPS34-IN1
DMSO
VPS34-IN1
17
LC3-I
LC3-II
55
αTubulin
-
-
+
+
4h BafA1

Fig. S4

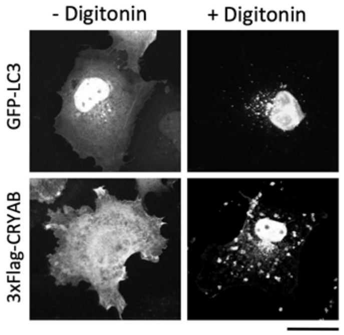

Fig. S5

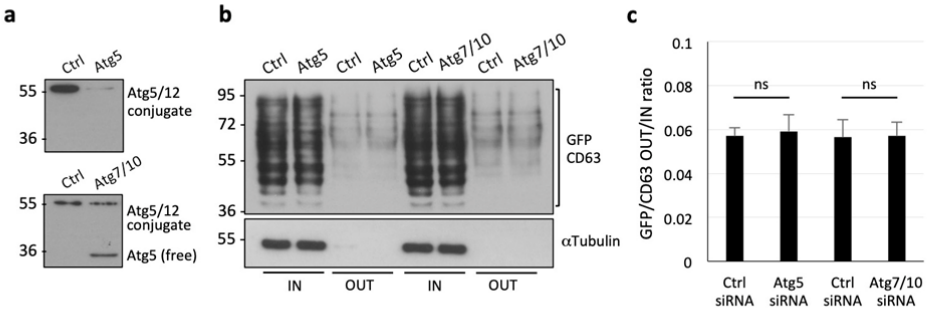

Fig. S6

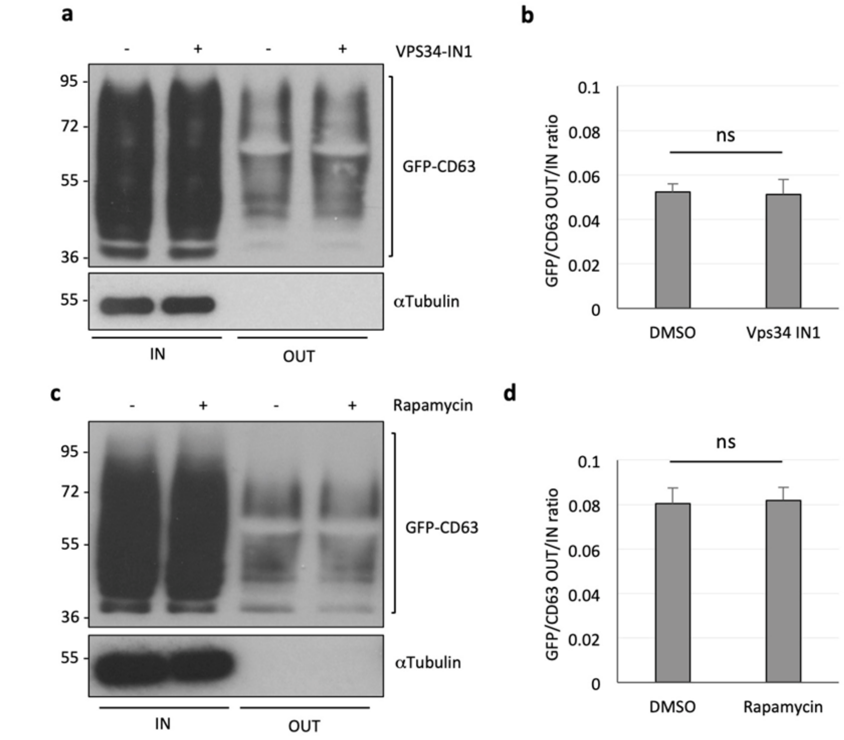

Fig. S7

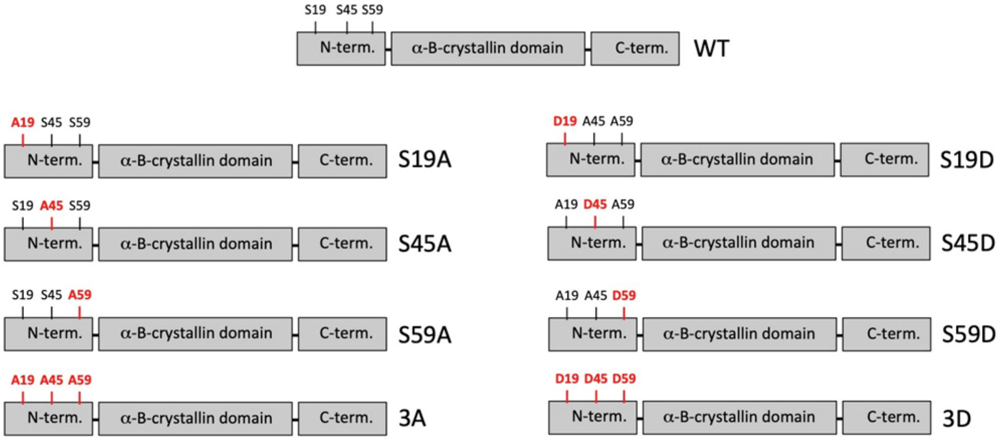
S19 S45 S59
N-term. α-B-crystallin domain C-term. WT
A19 S45 S59
N-term. α-B-crystallin domain C-term. S19A
S19 A45 S59
N-term. α-B-crystallin domain C-term. S45A
S19 S45 A59
N-term. α-B-crystallin domain C-term. S59A
A19 A45 A59
N-term. α-B-crystallin domain C-term. 3A
D19 A45 A59
N-term. α-B-crystallin domain C-term. S19D
A19 D45 A59
N-term. α-B-crystallin domain C-term. S45D
A19 A45 D59
N-term. α-B-crystallin domain C-term. S59D
D19 D45 D59
N-term. α-B-crystallin domain C-term. 3D

Fig. S8

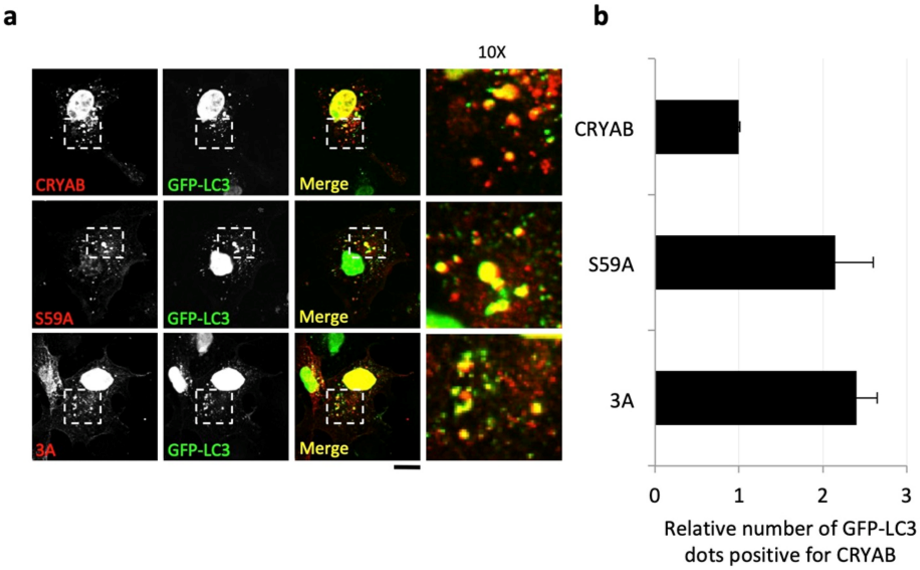
a
10X
CRYAB
GFP-LC3
Merge
S59A
3A
b
CRYAB
S59A
3A
0
1
2
3
Relative number of GFP-LC3 dots positive for CRYAB

Fig. S9

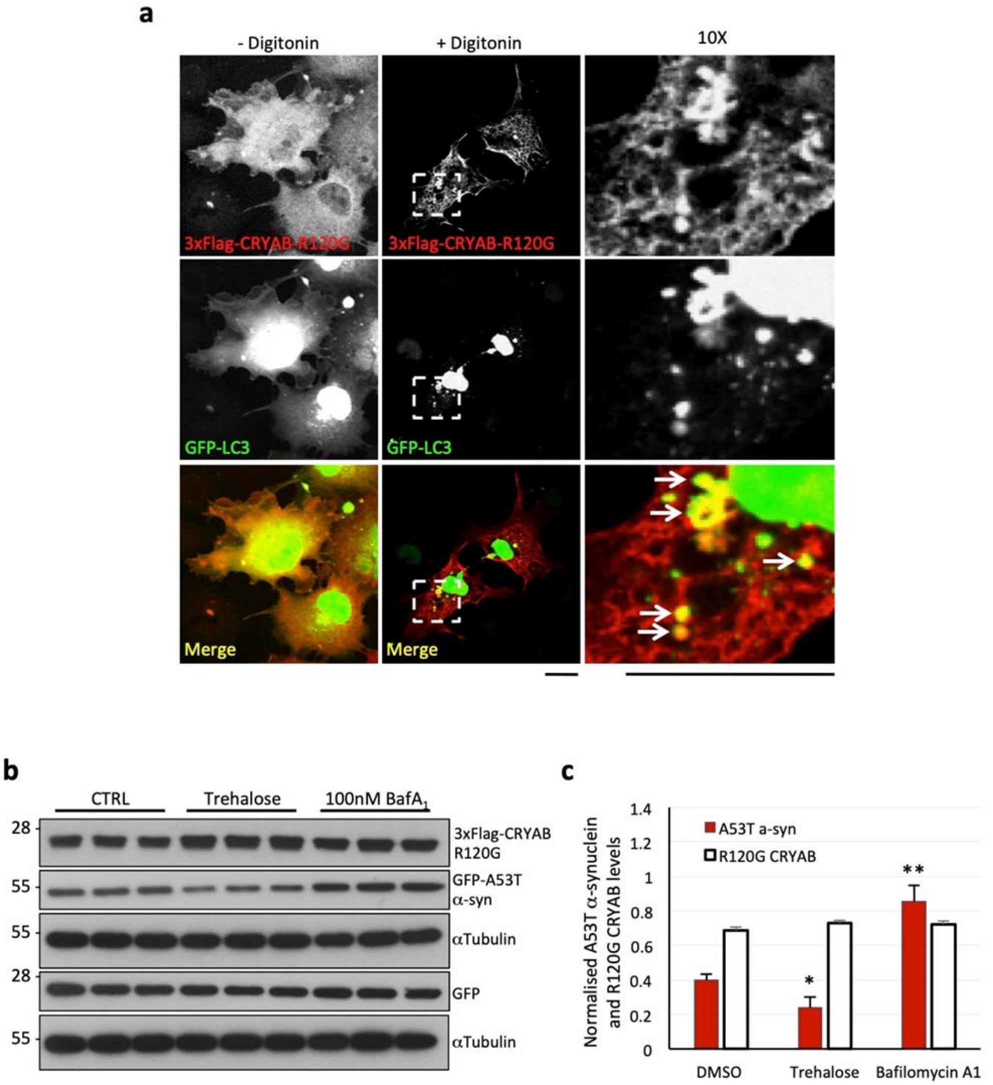
a
- Digitonin
+ Digitonin
10X
3xFlag-CRYAB-R120G
3xFlag-CRYAB-R120G
GFP-LC3
GFP-LC3
Merge
Merge
b
CTRL
Trehalose
100nM BafA1
28
55
55
28
55
3xFlag-CRYAB R120G
GFP-A53T α-syn
αTubulin
GFP
αTubulin
c
Normalised A53T α-synuclein and R120G CRYAB levels
A53T a-syn
R120G CRYAB
1.4
1.2
1
0.8
0.6
0.4
0.2
0
*
**
DMSO
Trehalose
Bafilomycin A1

FIGURE 1A

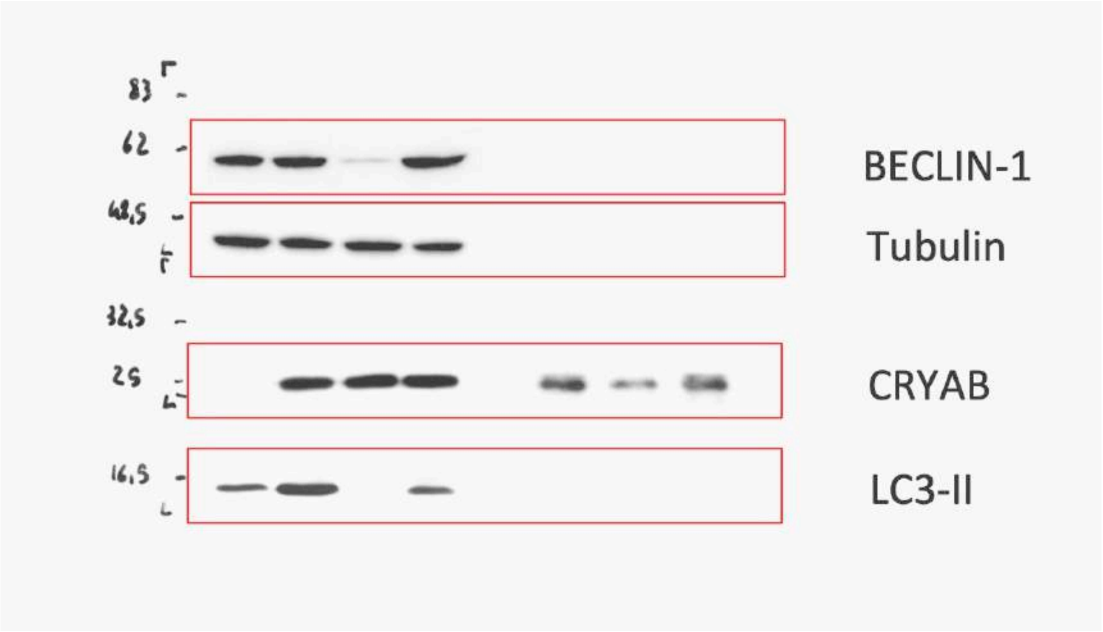

FIGURE 1B

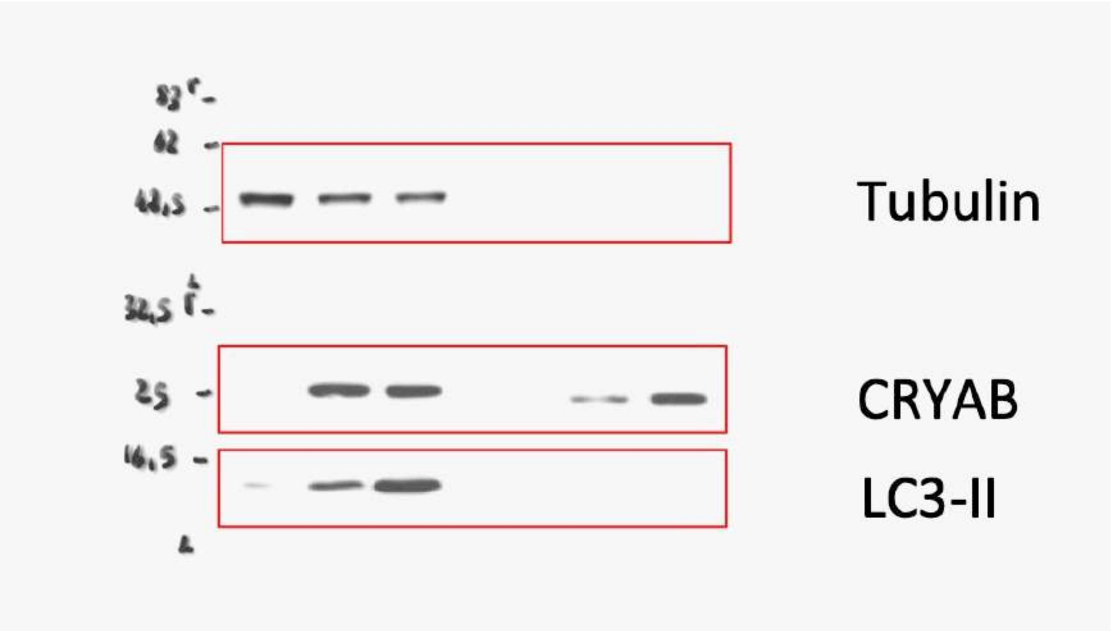

FIGURE 1C

Short exposure

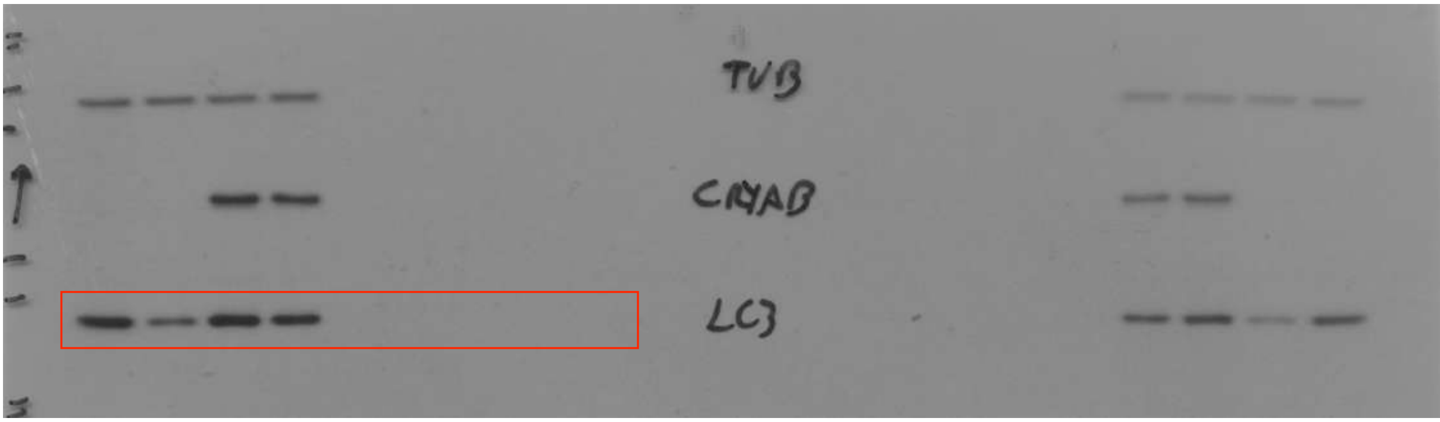

Medium exposure

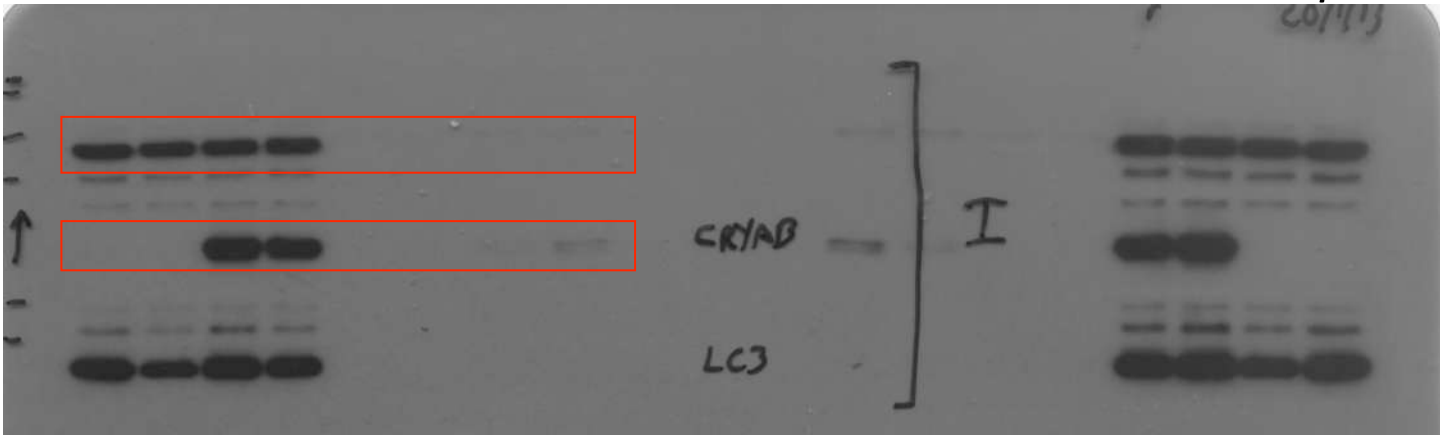

Long exposure

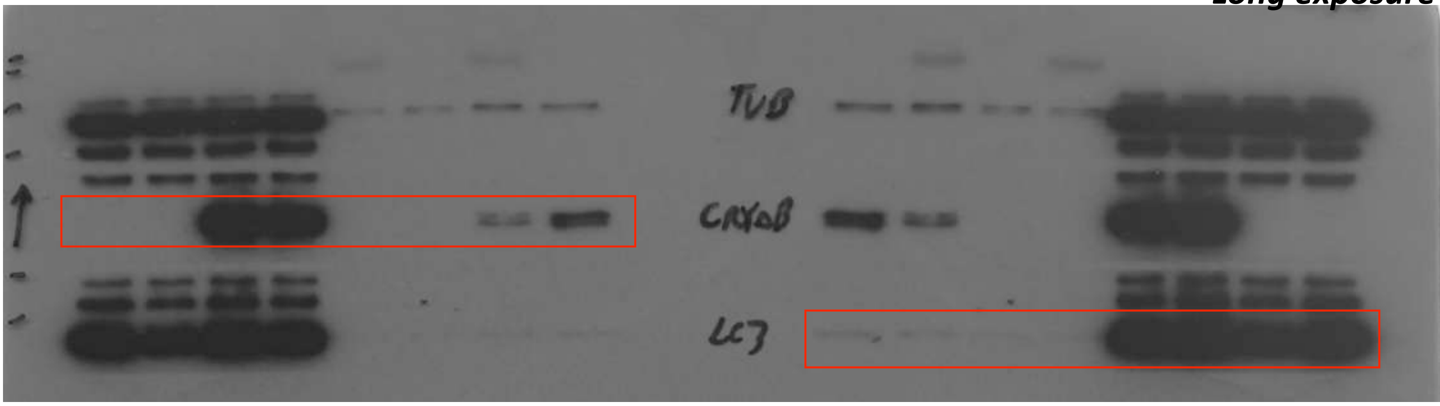

FIGURE 1G

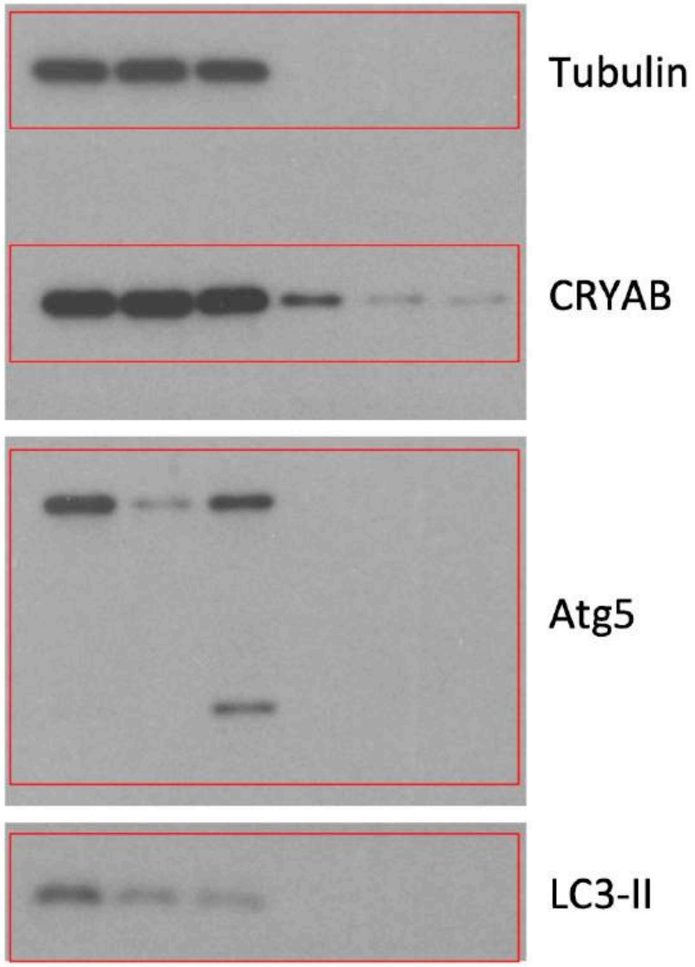

FIGURE 1J

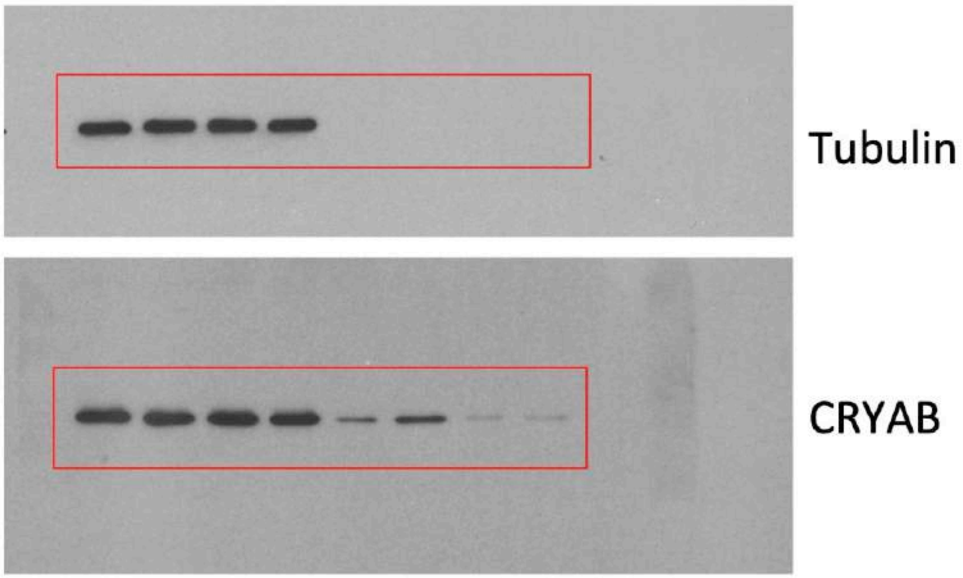

FIGURE 2A

Short exposure *Long exposure*

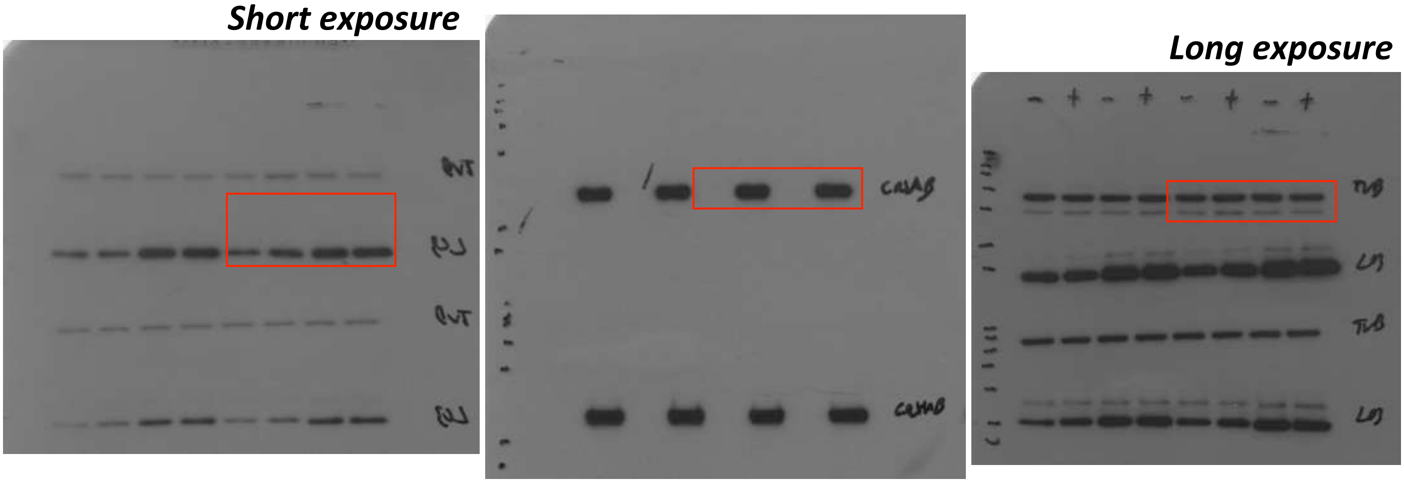

FIGURE 2C

Short exposure *Medium exposure* *Long exposure*

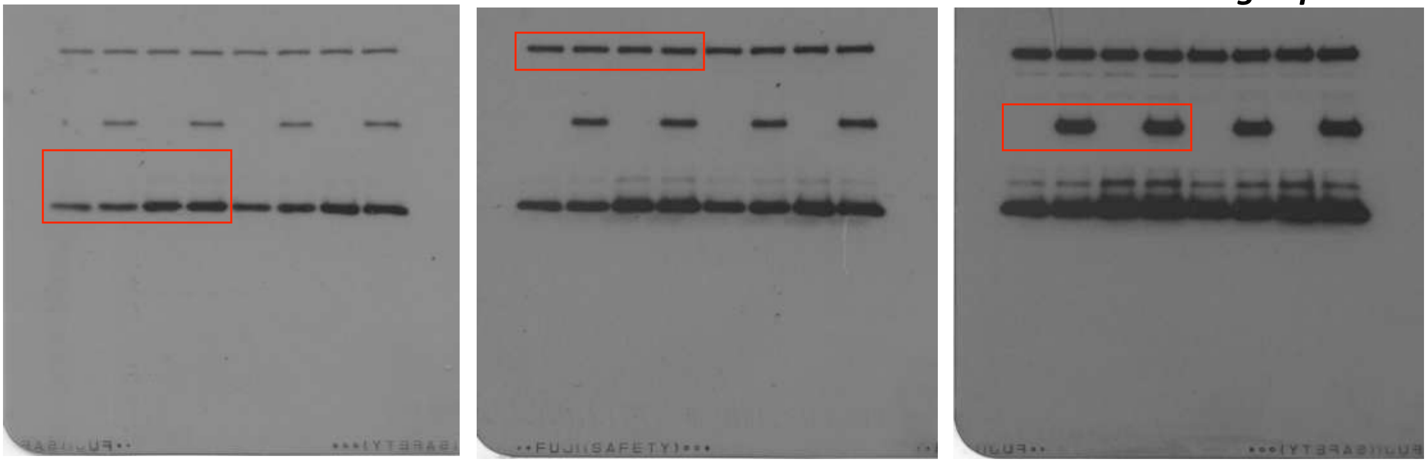

FIGURE 4A

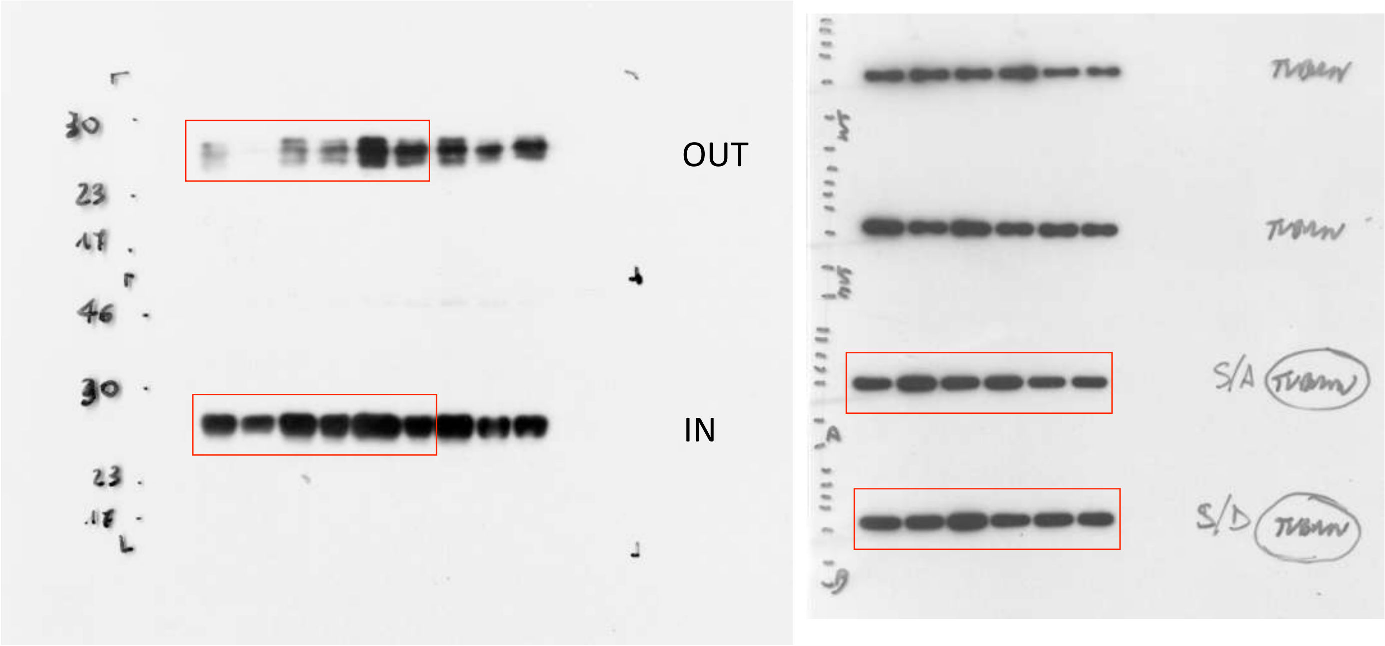

FIGURE 4B

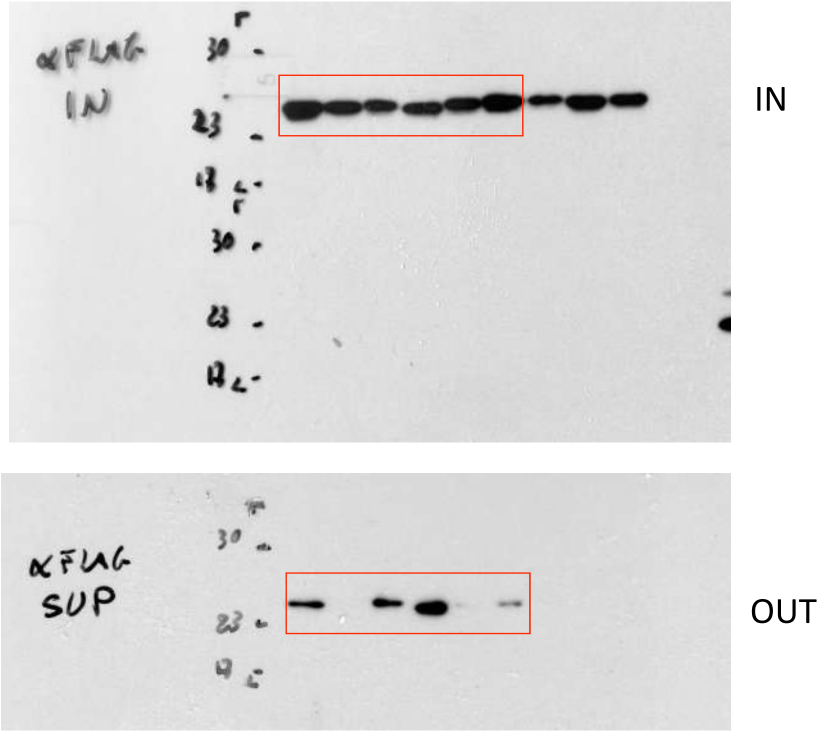

FIGURE S1

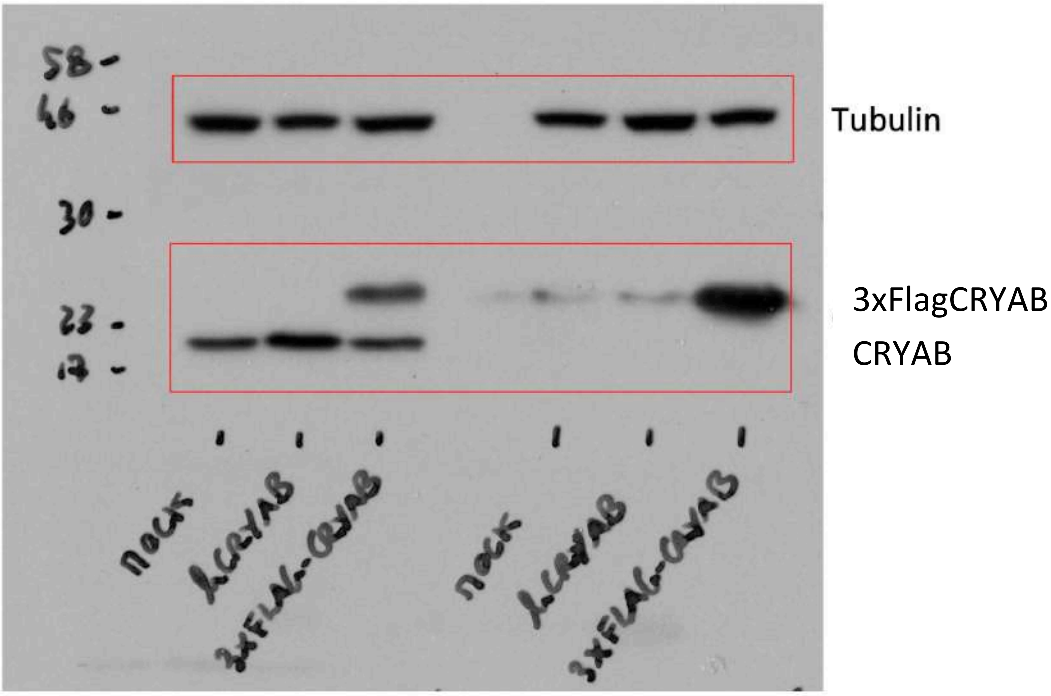

FIGURE S2A

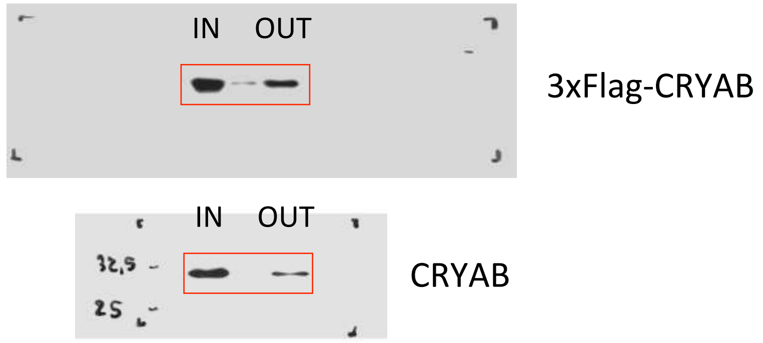

FIGURE S2B

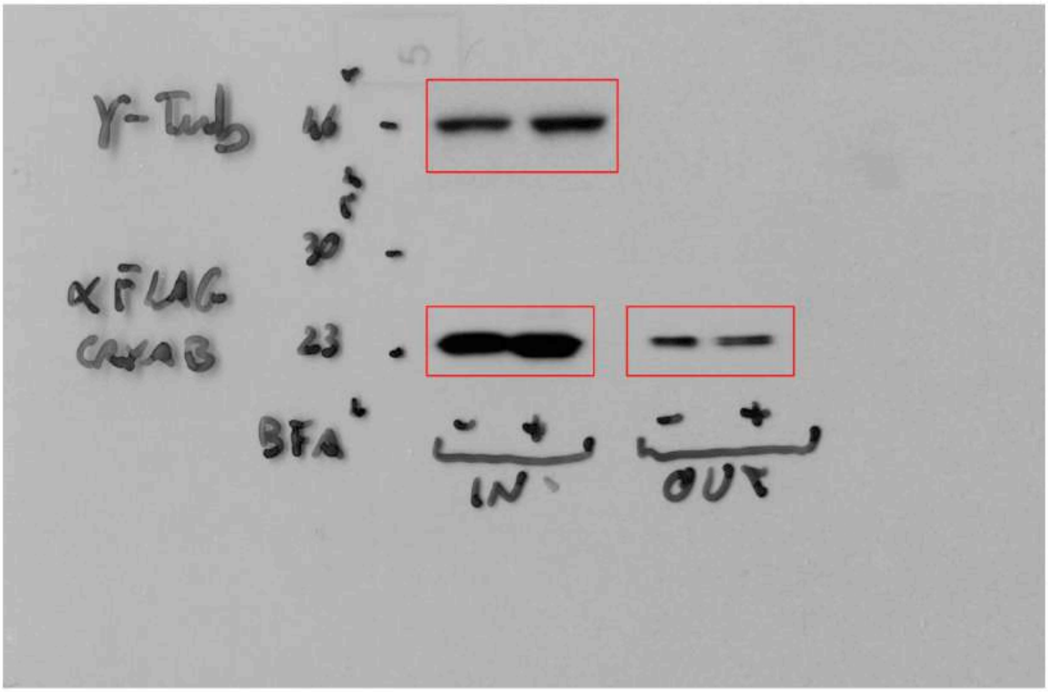

FIGURE S2C

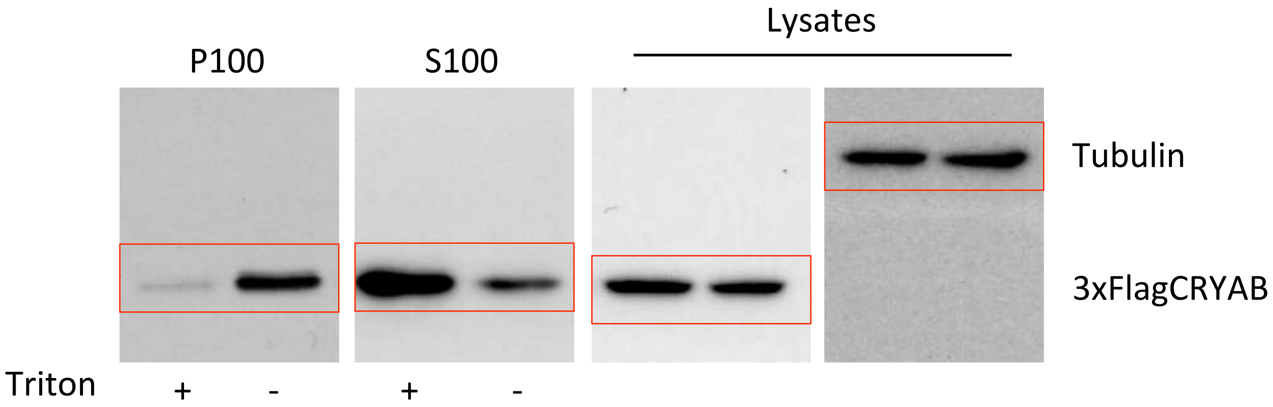

FIGURE S3

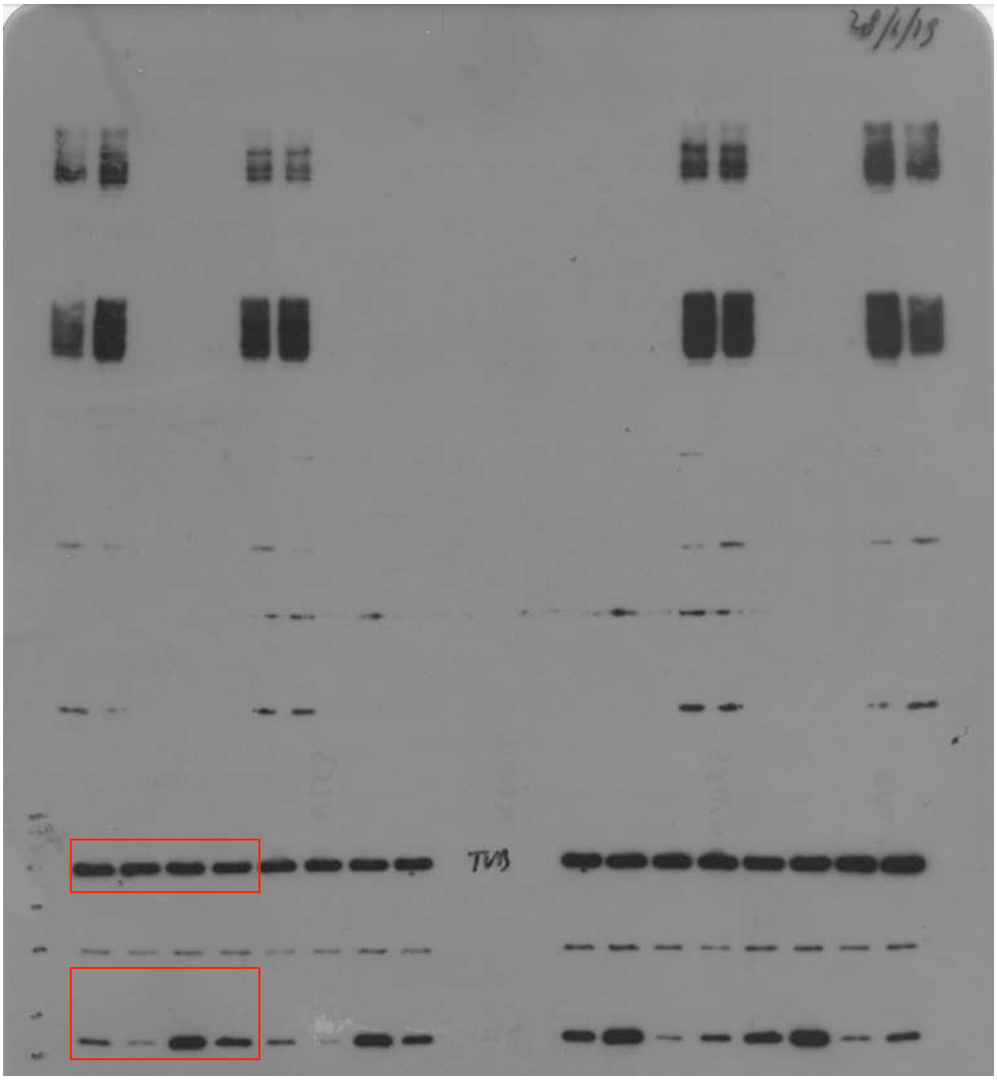

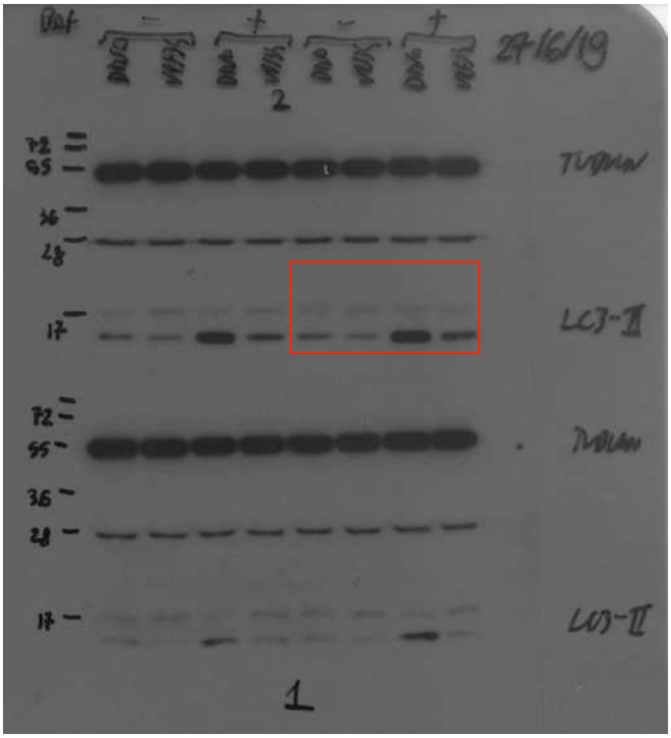

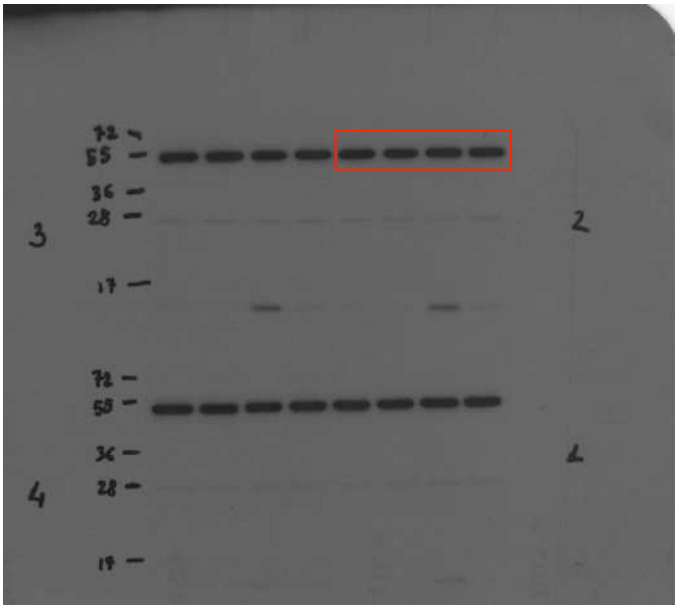

FIGURE S5

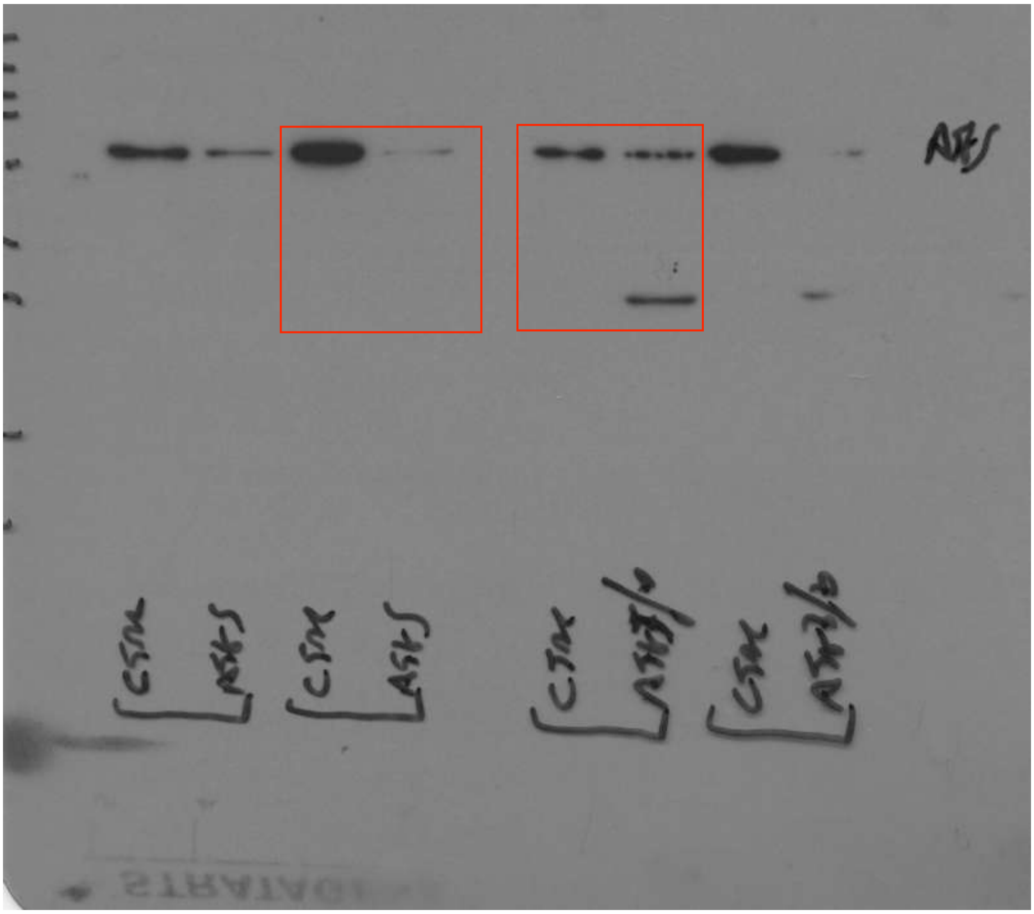

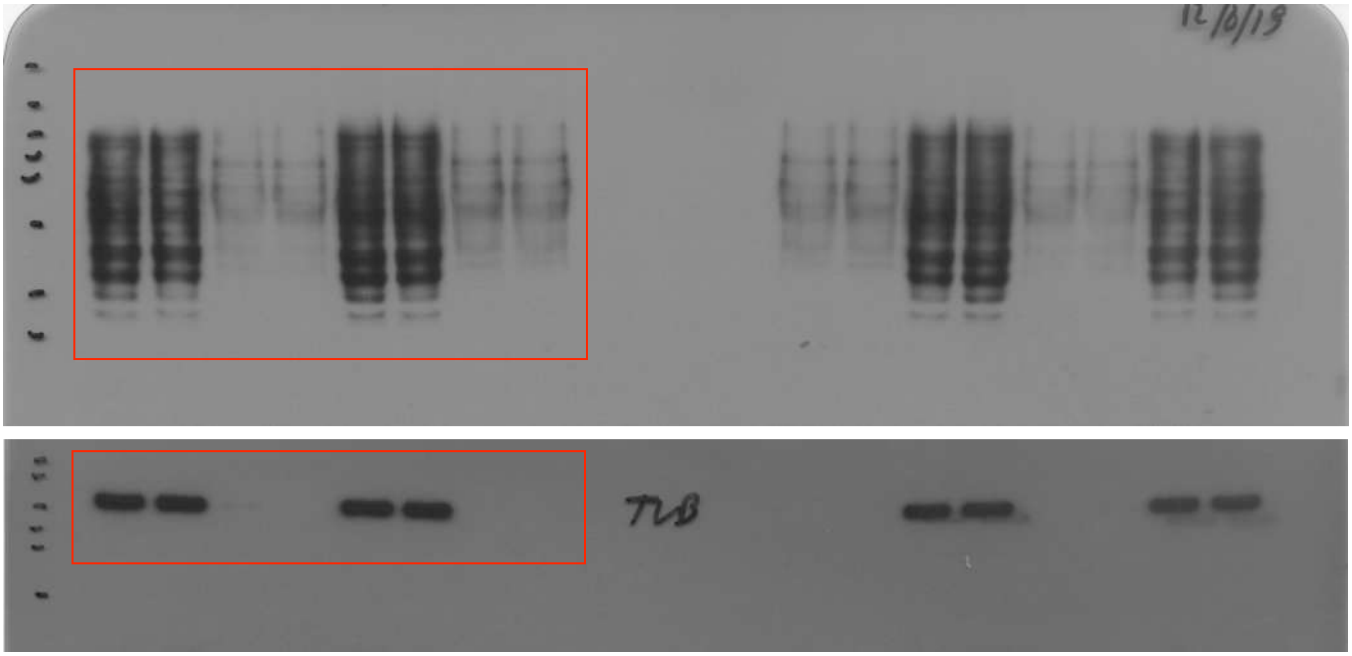

FIGURE S6

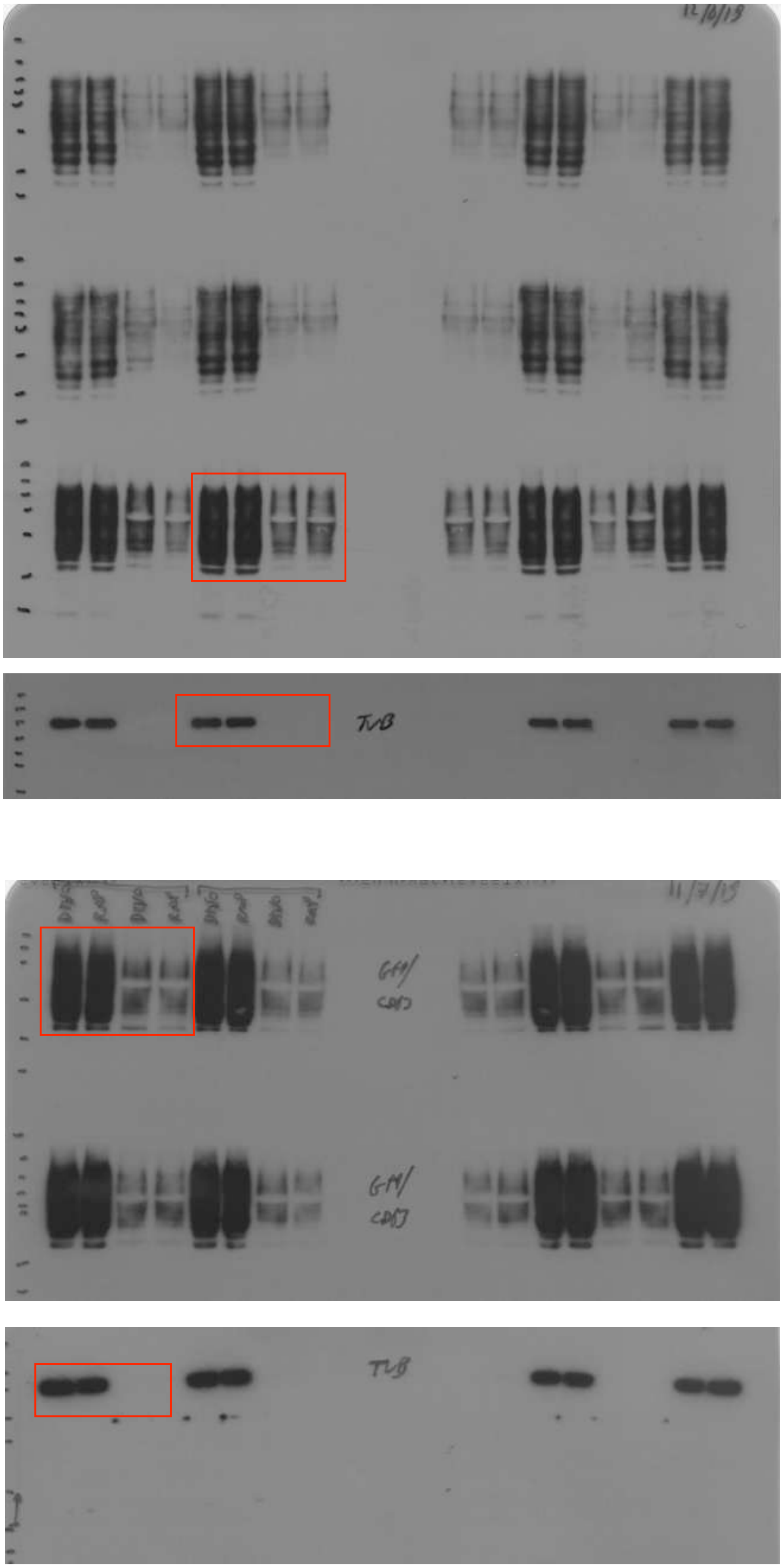

FIGURE S9

FLAG/R120G CRYAB

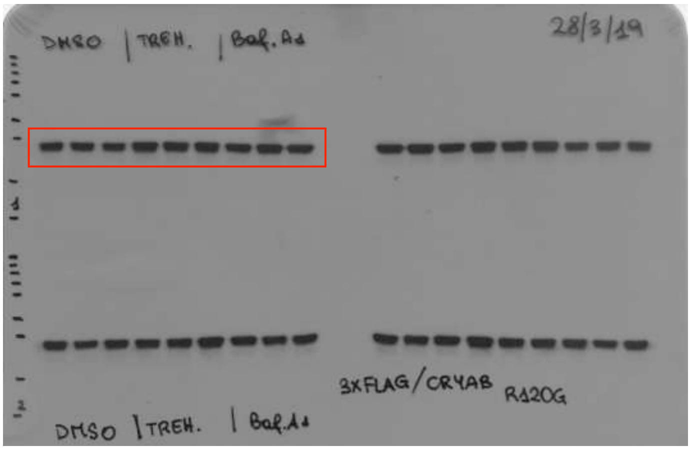

GFP/A53T α-syn

Empty-GFP

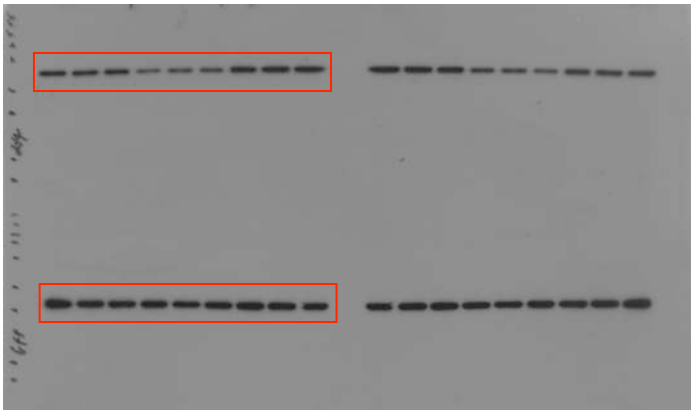

Tubulin

Tubulin

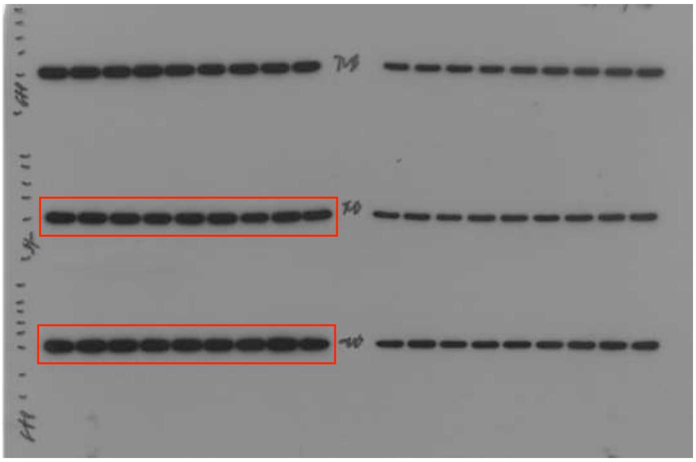

Supplement: Supplementary file 1 — Supplemenrtary informations [file 41598_2019_53226_MOESM1_ESM.pdf]
